# Aging Hallmarks and Progression and Age-Related Diseases: A Landscape View of Research Advancement

**DOI:** 10.1021/acschemneuro.3c00531

**Published:** 2023-12-14

**Authors:** Rumiana Tenchov, Janet M. Sasso, Xinmei Wang, Qiongqiong Angela Zhou

**Affiliations:** CAS, a Division of the American Chemical Society, 2540 Olentangy River Road, Columbus, Ohio 43202, United States

**Keywords:** Aging, longevity, epigenetic, senescence, inflammaging, telomere, stem cell, brain aging

## Abstract

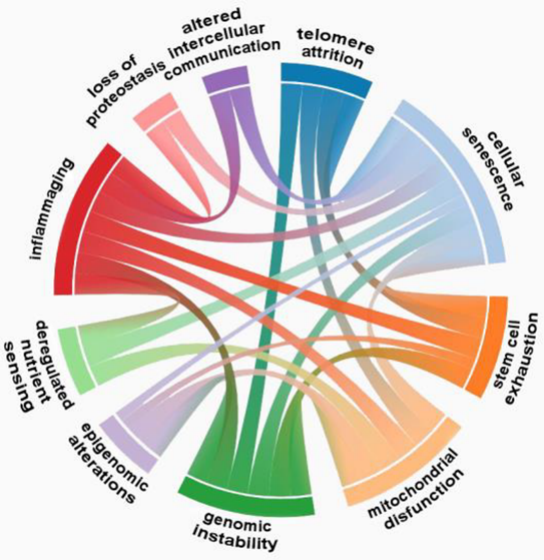

Aging is a dynamic, time-dependent process that is characterized by a gradual accumulation of cell damage. Continual functional decline in the intrinsic ability of living organisms to accurately regulate homeostasis leads to increased susceptibility and vulnerability to diseases. Many efforts have been put forth to understand and prevent the effects of aging. Thus, the major cellular and molecular hallmarks of aging have been identified, and their relationships to age-related diseases and malfunctions have been explored. Here, we use data from the CAS Content Collection to analyze the publication landscape of recent aging-related research. We review the advances in knowledge and delineate trends in research advancements on aging factors and attributes across time and geography. We also review the current concepts related to the major aging hallmarks on the molecular, cellular, and organismic level, age-associated diseases, with attention to brain aging and brain health, as well as the major biochemical processes associated with aging. Major age-related diseases have been outlined, and their correlations with the major aging features and attributes are explored. We hope this review will be helpful for apprehending the current knowledge in the field of aging mechanisms and progression, in an effort to further solve the remaining challenges and fulfill its potential.

## 1 Introduction

The growing social and economic concern of an aging world population has catapulted aging-related research into the spotlight. Indeed, over the past decades, progress in medicine has powered a significant increase in life expectancy worldwide. Thus, more than 2 billion individuals are expected to be older than the age of 60 by 2050.^1^ This demographic milepost will come with a major increase in age-related diseases, such as Alzheimer’s disease, cardiovascular disorders, and cancer, which effectively double in incidence every 5 years passing the age of 60.^2^ The relationship between aging and these diseases has triggered fundamental research into the aging mechanisms and approaches to attenuate its effect.

Aging is broadly defined as a gradual functional decline in the living organism’s intrinsic ability to defend, maintain, and repair itself in order to keep working efficiently and has attracted attention throughout the history of civilization.^3,4^ The health and survival of an organism present a dynamic equilibrium between the processes of damage and repair, alteration, and maintenance, a conventional concept of which is homeostasis. This concept, recently replaced by homeodynamics, involves the constantly changing interrelations of body constituents while an overall equilibrium is maintained.^5^ Thus, aging is characterized as a multidimensional process involving a gradual contraction of homeodynamic space. It affects many different aspects of life including physical health, cognitive functioning, emotional well-being, and social relationships. There is a consensus that aging is associated with two key aspects: (i) the progressive decline of numerous physiological processes, such as the body’s ability to accurately regulate homeostasis, and (ii) the enhanced risk of developing severe diseases such as cancer or cardiovascular disease. However, while aging is a major risk factor for many chronic diseases, it is important to recognize that aging and disease are not synonymous. Many older adults maintain good physical and mental health well into old age, and there is growing interest in promoting “successful aging” by focusing on factors that contribute to overall health and well-being.^6,7^

Researchers have distinguished between two categories of age: the chronological age based on the birthdate, and the biological age, which measures the true age at which the cells, tissues, and organs appear to be, based on biochemistry.^8^ While it is impossible to alter the chronological age, there are ways to manage biological age. Since aging is influenced by multiple factors, including genetics, lifestyle aspects such as diet, exercise, and stress, environmental factors such as pollution and climate change, and social factors such as social support and socioeconomic status, interventions such as lifestyle adjustments, medical treatments, and social programs can help promote healthy aging and extend the lifespan. Understanding the complex interactions between these factors is essential for promoting healthy aging.^9^

Along with the whole organism, the functional capabilities of the brain gradually degrade upon aging, manifesting as declines in learning and memory, attention, decision-making capacity, sensory perception, and motor management. The aging brain exhibits significant indicative signs of impaired bioenergetics, weakened adaptive neuroplasticity and resilience, anomalous neuronal network activity, dysfunctional neuronal calcium homeostasis, accumulation of oxidatively modified molecules and organelles, and inflammation.^10−16^ Reduced number and maturity of dendritic spines in aging organisms, along with alterations in synaptic transmission, may indicate abnormal neuronal plasticity directly related to impaired brain functions.^14^ At worst, neurodegenerative and cerebrovascular diseases, which strongly damage the basic functions of the brain, may develop. Thus, age-associated alterations render the aging brain vulnerable to degenerative disorders including Alzheimer’s and Parkinson’s diseases, stroke, and various kinds of dementia.^17,18^ While currently there is no cure for these age-related brain disorders, early detection by recognizing symptoms can help slow the progression of the disease.

In fact, most vital organs and tissues of the body undergo a certain age-related decline in function. Thus, muscle strength decays with age, bones weaken losing mass and/or density, and skin exhibits visible changes and also may show signs of impaired wound healing. Blood levels of certain hormones (e.g., growth hormone, androsterone, testosterone) decline with age, while others (e.g., gonadotropins, insulin) increase with age. Overall immune function deteriorates, becoming slower to respond, leading to an increased susceptibility to various infectious diseases. Sleep worsens and certain sleep disorders develop. Vision and hearing decline. Kidney tissue decreases and kidney function diminishes, along with multiple other age-related changes.^7,19−21^

The attributes of aging include a variety of interconnected molecular and cellular mechanisms that act jointly to control the aging process.^22^ Thus, aging has been characterized as a progressive degenerative status accompanied by processes like stem cell exhaustion, tissue inflammation, extracellular matrix modifications, cellular senescence, and metabolic dysfunction.^23^ These cellular and tissue modifications reflect inherent molecular deviations in mitochondria, epigenetics, DNA maintenance, proteostasis, intercellular interactions, and nutrient sensing, which give rise to genomic instability and impairment, including telomere dysfunction.^23^

**Figure 1:**
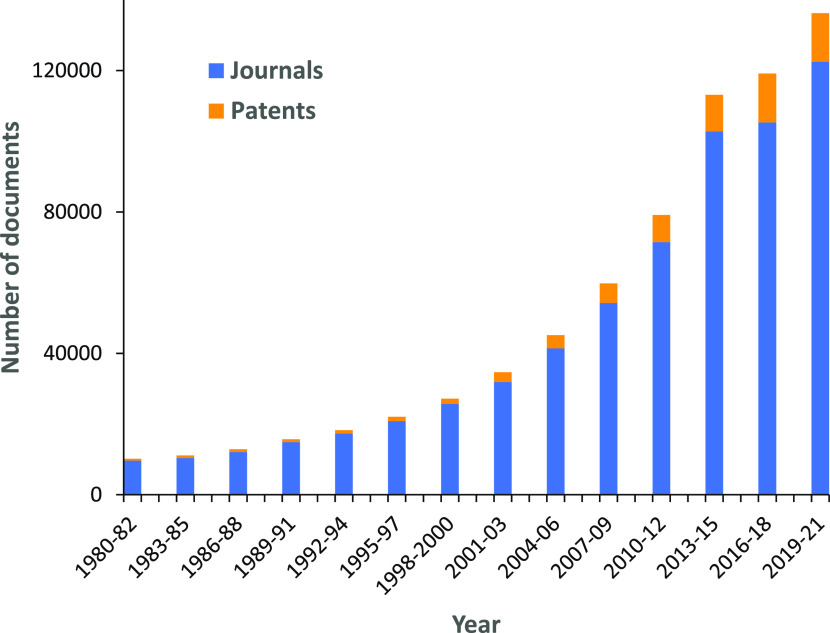
Yearly growth of the number of aging-related documents (journal articles and patents) in the CAS Content Collection.

The research focused on aging mechanisms and attributes has undergone a steady growth, especially intense in the past decade (Figure 1). It has brought the understanding that although aging is not by itself a disease, it is the major risk factor for developing many severe and chronic diseases such as cancer, cardiovascular diseases, and neurodegenerative diseases such as Alzheimer’s disease. Furthermore, many diseases seem to accelerate the aging process, manifested as declines in functionality and reduced quality of life. This insight has brought the rapidly growing field of aging research to the forefront, with the major challenge to develop a distinct understanding of the basic biology underlying changes that accompany aging by identifying genetic, molecular, and cellular factors that control the rate of aging processes.

Multiple studies have attained notable knowledge of how aging takes place and how it is controlled by cellular and molecular mechanisms. Factors influencing the aging process and longevity have been identified including telomere shortening, mitochondrial dysfunction, oxidative stress, deregulated nutrient-sensing, DNA repair deterioration, DNA damage, protein homeostasis alterations resulting in accumulation of misfolded proteins, and changes in epigenetic control.^24−27^

In this paper, we review advances in research on aging hallmarks and factors. We examine data from the CAS Content Collection,^28^ the largest human-compiled collection of published scientific information, and analyze the publication landscape of recent research in this area in an effort to provide insights into the research advances and developments. We review the current concepts related to the major aging hallmarks on the molecular, cellular, and organismic level and age-associated diseases, as well as the major biochemical processes associated with aging. The review is intended to guide readers in envisioning the current knowledge in the research field related to aging mechanisms and dimensions, for evaluating challenges and growth opportunities, in an effort to further achieving its potential.

## 2 Mechanisms and Physiology of Aging

Aging is typified by a gradual loss of physiological fitness, leading to deteriorated functions and an enhanced vulnerability. Such deterioration is the key risk factor for critical pathologies such as cancer, diabetes, cardiovascular and neurodegenerative disorders, and many other maladies. Various studies have examined how aging takes place and how it is controlled by sophisticated cellular and molecular mechanisms at different periods of life.^25,26^ Multiple factors involved in the aging process and longevity have been described. The finding that the rate of aging is regulated, at least to a certain extent, by genetic routes and biochemical processes conserved in evolution has triggered remarkable advances in aging research. Aging is basically damage that accumulates over time and is manifested in certain physiological forms, considered as the hallmarks of aging.^23,24,29−32^

A hallmark of aging is a distinct attribute that occurs upon normal aging and correlates with the decline in biological function and increased risk of age-associated diseases. Moreover, in order to qualify as a hallmark, the attribute needs to play a causative role in the process of aging.^23,31^ The definition of nine molecular and cellular hallmarks of aging in 2013 provided a background framework to channel future aging research.^23^ These hallmarks included the following:

- Genomic instability: It refers to the accumulation of DNA damage and mutations over time, which can lead to a variety of age-related diseases such as cancer and neurological disorders.
- Telomere attrition: Telomeres are the protecting caps at the chromosome ends, which shorten with each cell division. This process is associated with cellular senescence, a state of permanent growth arrest that can contribute to aging.
- Epigenetic alterations are changes in gene expression patterns that are not caused by changes in the DNA sequence itself. These changes can contribute to the development of age-associated diseases.
- Loss of proteostasis refers to the gradual breakdown of protein homeostasis or the ability of cells to maintain their proper protein folding and degradation.
- Dysregulated nutrient sensing: The body’s ability to sense and respond to changes in nutrient availability becomes impaired with age, which can lead to metabolic dysfunction and an increased risk of age-associated diseases such as diabetes.
- Mitochondrial dysfunction: Mitochondria are the powerhouses of the cell and play a critical role in energy production. With age, mitochondrial function can decline, leading to decreased energy production efficiency and more oxidative stress, therefore an increased risk of age-related diseases.
- Cellular senescence: As mentioned earlier, cellular senescence is a status of permanent growth arrest that can contribute to the aging process.
- Stem cell exhaustion: Stem cells have the capability to differentiate into various different cell types and play a critical role in tissue repair and regeneration. With age, the number and function of stem cells can decline, leading to decreased tissue repair and an increased risk of age-related diseases.
- Altered intercellular communication: The process of aging implicates alterations at the level of intercellular interaction, including endocrine, neuroendocrine, or neuronal communication. Thus, neurohormonal signaling is likely to be deregulated in aging thereby modifying the mechanical and functional properties of all tissues.

**Figure 2:**
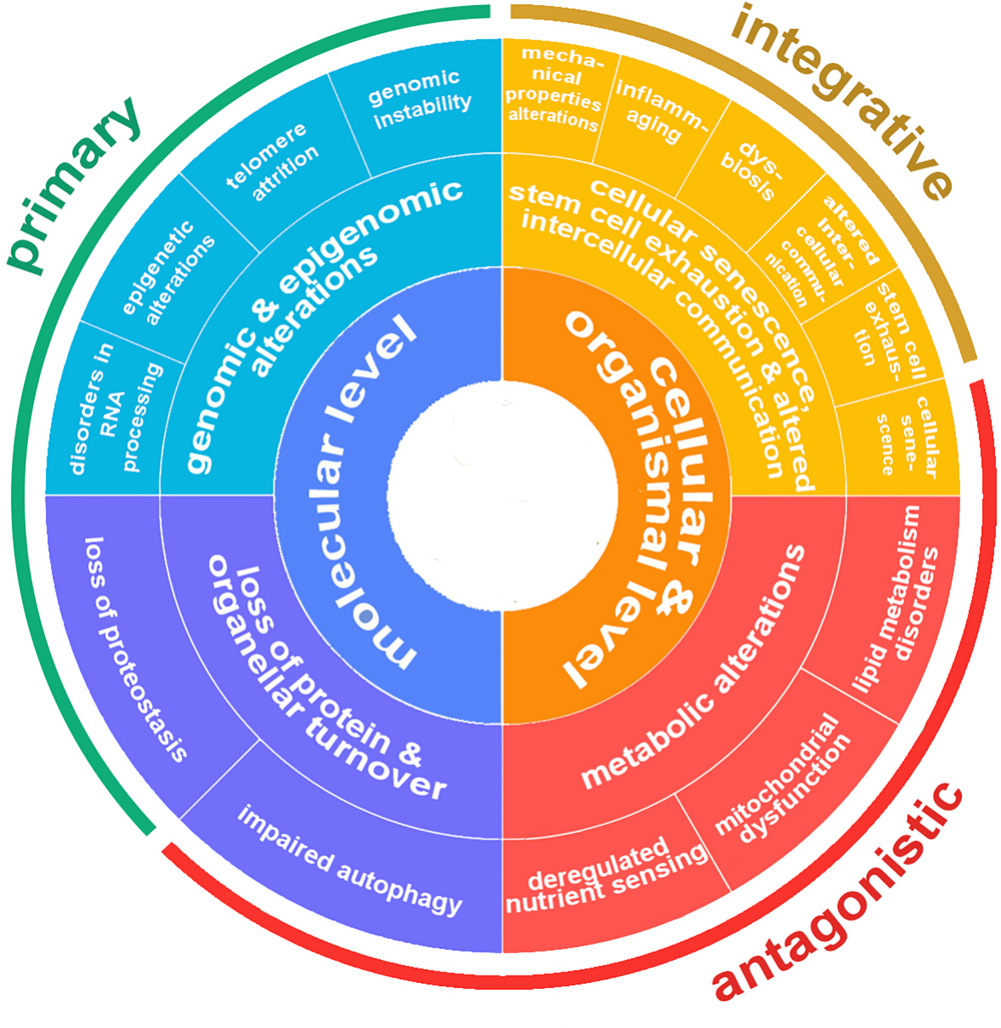
Scheme of the currently identified hallmarks of aging along with their classification.

The first four hallmarks (genomic instability, telomere attrition, epigenetic alterations, and loss of proteostasis) have been classified as primary hallmarks because they are the primary causes of cellular damage (Figure 2).^23,32^ They are all, unequivocally, negative. The next three hallmarks (dysregulated nutrient sensing, mitochondrial dysfunction, and cellular senescence) are classified as antagonistic because they are related to the responses to the primary hallmarks. Contrary to the primary hallmarks, antagonistic ones have multidimensional effects. Initially these responses mitigate the damage caused by the primary hallmarks but eventually become harmful themselves. For example, cellular senescence, or cell cycle arrest, can protect the organism from cancer but also promote aging. The last two hallmarks (stem cell exhaustion and altered intercellular communication) have been characterized as integrative since they take place when the accumulated damage, resulting from the primary and antagonistic hallmarks, cannot be compensated by the cellular homeostatic mechanisms and reparative processes.^23,32^ Integrative hallmarks further promote the deterioration of cells that are responsible in due course for aging. Both of these hallmarks influence tissue homeostasis and function.

As a result of the scientific research progress, additional aging attributes have been identified with time. A decade after the initial nine hallmarks were suggested, an additional five aging characteristic features surfaced,^31,33^ which are the following:

- Compromised autophagy impeding the clearance of misfolded proteins is observed in numerous aging conditions including neurodegeneration and immunosenescence.
- Microbiome disturbances (dysbiosis) that indicate in particular alterations in microbial populations and loss of species diversity, which, along with the age-related loss of structural integrity of the guts, can drive inflammation.
- Splicing dysregulation, associated with changes in the regulatory splicing factors, may be a significant contributor to the development of cellular senescence.
- Chronic low-level inflammation (inflammaging) is a widespread feature of aging, taking place in the absence of explicit infection, representing a substantial risk factor for morbidity and mortality in the elderly.
- Mechanical properties alterations occur both in cells and in the extracellular environment and can lead to multiple age-related diseases such as hypertension and to accelerated aging in patients with diabetes due to glycation cross-linking between collagen molecules.

Recently, additional mechanisms of aging related to lipid metabolism have been implied:

- Accumulation of sphingolipids. Ceramides, a common class of sphingolipids, accumulate in aging muscle and reduce its function, impacting the functional capacity of older adults.^34−36^
- Dysregulation of cholesterol metabolism. Senescent cells pile up cholesterol in the lysosomes to support the senescence-associated secretory phenotype (SASP).^37^

Furthermore, multiple other common physiological features of aging have been identified and examined in research publications:

- Decline in immune function: Upon aging, the immune system becomes less effective at fighting off infections and diseases, which can lead to increased risk of infections and certain types of cancer.^38,39^
- Hormonal changes: The levels of many hormones in the body change upon aging, e.g., the levels of growth hormone, testosterone, and estrogen tend to decrease, while levels of cortisol (the stress hormone) tend to increase.^40−42^
- Changes in body composition: With age, there is a tendency to lose muscle mass and gain fat. This can increase the risk of metabolic disorders such as type 2 diabetes and cardiovascular disease.^43−45^
- Decreased cognitive function: Cognitive decline has been widely observed upon aging, particularly in areas such as memory and processing speed.^46−48^
- Increased risk of falls and fractures: With aging, bones become weaker and balance may decline, increasing the risk of falls and fractures.^49−51^

In the following, all of these hallmarks of aging are discussed in detail.

### 2.1 Genomic and Epigenomic Alterations

#### 2.1.1 Genomic Instability

Accumulation of genetic impairment over a lifetime is a common feature of aging. Indeed, the human genome is under continual challenges by DNA-destructive attacks that endanger cellular homeostasis. These include various exogenous physical, chemical, and biological threats such as viruses, UV damage, and chemicals, as well as endogenous hazards such as DNA replication inaccuracies, spontaneous hydrolytic reactions, and reactive oxygen species (ROS) (a detailed description of oxidation as one of the major aging-related biochemical processes is provided in section 2.6).^52^ Thus, somatic mutations of the nuclear DNA accumulate within cells of aged organisms.^53^ Mutations and deletions in mitochondrial DNA may also contribute to aging.^54^ Accumulated defects in the nuclear lamina are another possible source of genomic instability, except for the genomic damage affecting nuclear or mitochondrial DNA.^55^ Further, cell cycle stress, alterations in gene expression, and gene regulation take place as a direct consequence of genomic instability. Eventually, it results in age-related cellular degeneration and functional decay. Aging and degenerative disease happen as the ultimate outcome of genomic instability.^56^ Thus, there is extensive evidence that genomic damage accompanies and is causatively related to aging. The produced genetic damages are diverse, including impairments such as point mutations, translocations, telomere shortening, and others.^23^ To counteract these DNA damages, organisms have developed repair mechanisms such as specific processes for maintaining the appropriate length and functionality of telomeres and for ensuring the integrity of mitochondrial DNA.^57−59^

#### 2.1.2 Telomere Attrition

**Figure 3:**
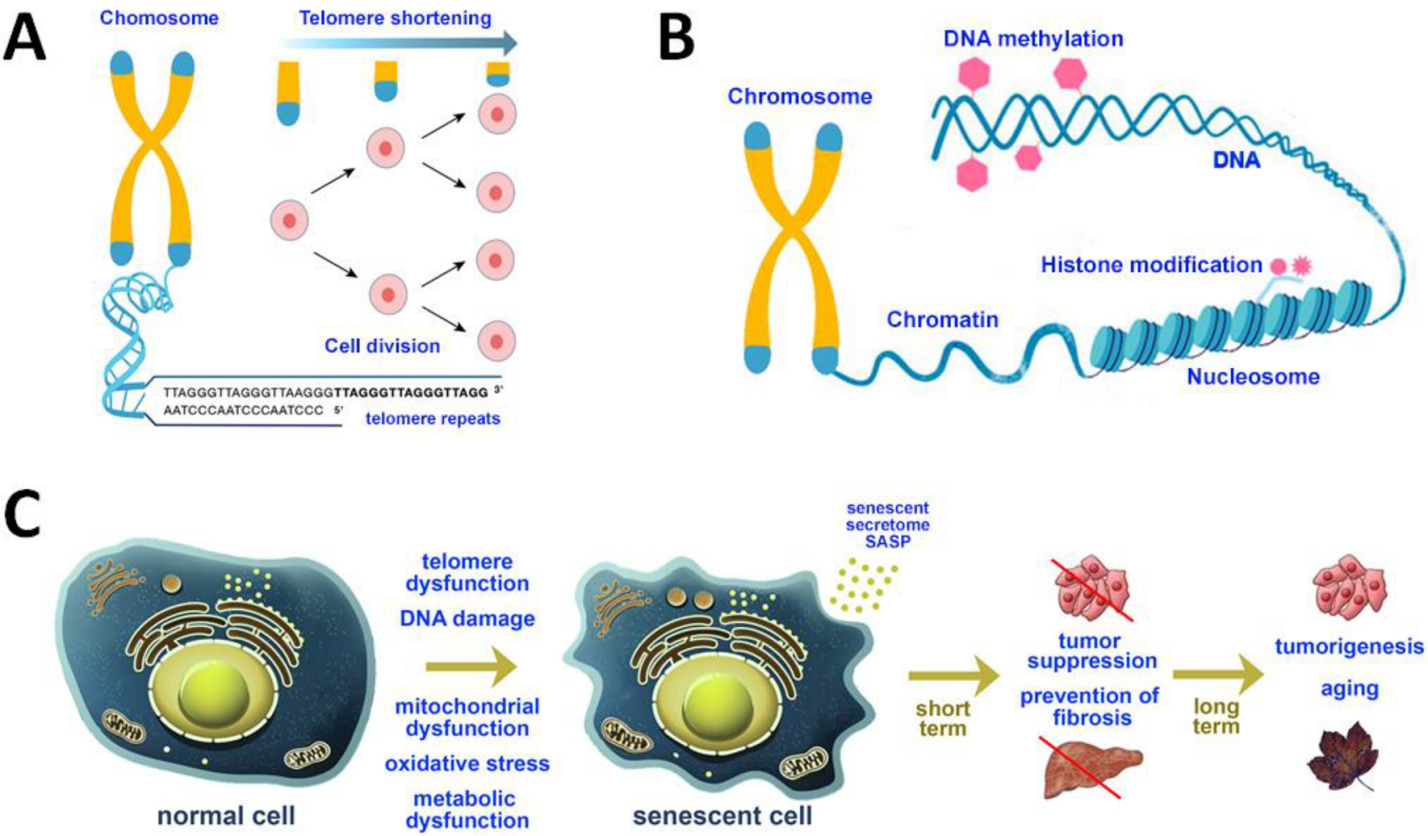
(A) Scheme of the structures of chromosomes and telomeres. At their ends, chromosomes exhibit repeated base segments called telomeres, which truncate with each replication cycle. Telomeres are known to shorten upon cell division, as a result of incomplete replication. (B) Scheme of the common epigenetic alterations including DNA methylation and histone modifications. (C) Schematic presentation of normal cells and senescent cells, secreting a senescence-associated secretory phenotype (SASP). In the short term, senescence growth arrest prevents tumorigenesis and fibrosis. The loss of proliferative capacity, which accompanies senescence, impairs tissue regeneration and stimulates aging. SASP can promote tumor growth and progression by stimulating angiogenesis and extracellular matrix remodeling.

Although DNA damage accumulation with age involves the genome generally, certain regions of the chromosomes, such as telomeres, are especially vulnerable to age-associated deterioration (Figure 3A).^58,60^ Telomeres are chromatin structures at the distal ends of chromosomes, including conserved microsatellite repeats TTAGGG, which cap and protect the end of a chromosome from recombination and degradation.^61^ They allow the chromosome to replicate properly during cell division. The telomere length in humans at birth is ∼10 000–19 000 base pairs.^62^ They are known to shorten during cell division as a result of imperfect replication, losing ∼50–200 base pairs per cell division.^63−65^ Such gradual telomere shortening restricts the number of times that a cell can divide. It is considered to act as a “molecular clock” correlated to organism aging.^66,67^ Telomere length is one of the biomarkers of aging and biological age. Specifically, telomere shortening below a critical length causes telomere protection deficiency, chromosomal instability, and reduced cell viability. This excludes germ cells and certain cancerous cells which are known to express high levels of telomerase, thus avoiding significant telomere shortening and supporting cell viability.^60,67^

Telomerase is a specialized DNA polymerase with the ability to replicate the distal ends of DNA molecules. Telomerase is highly expressed in embryonic stem cells; however, it is not expressed in most mammalian somatic cells, which results in cumulative loss of telomere-shielding sequences at the chromosome ends.^68^ Such telomere exhaustion explicates the limited proliferation ability of some cells cultured *in vitro*, the so-called Hayflick limit specified as the number of times a normal somatic cell population will keep dividing until cell division halts.^69^ Studies on genetically modified animals have reported causative relationships between telomere loss, cellular senescence, and aging, indicating that aging can be reverted by telomerase activation.^23^ Thus, normal aging is associated with telomere attrition, while pathological telomere dysfunction accelerates aging.^70^

Telomeres are particularly predisposed to age-associated deterioration because of the existence of complexes termed shelterins.^71,72^ The key function of shelterins is to form T-loops at the ends of chromosomes, which safeguard telomeres by avoiding them being identified as DNA damage by the DNA polymerase. This has the undesirable effect thought of making it difficult for DNA polymerase to repair telomere damage.^23^ Thus, both extending telomeres and developing a mechanism to repair DNA damage in telomeres are required in order to deal with this aging pathway.

#### 2.1.3 Epigenetic Alterations

Although a great amount of research has been devoted to the genetic factors that directly affect aging, nongenetic control of aging has gained considerable interest lately as an important aspect in raising awareness of the process of aging. Nongenomic modifications that affect gene expression and modify the chromatin structure are referred to as epigenetic alterations and are generally defined as alterations in genomic regulation not directly encoded by DNA, i.e., alterations that do not change the DNA sequence but instead control gene operations.^73^ Such changes take place when methyl groups are added to or removed from DNA (DNA methylation/demethylation). Methylation is a process in which a methyl group (−CH_3_) is attached to a cytosine base (C) of DNA. It initiates DNA condensation, a configuration in which genes have not been transcribed. Methylation levels change throughout life but generally tend to decrease upon aging. Epigenetic alterations take place also when post-translational modifications are made to the histones and upon chromatin remodeling (Figure 3B) (a detailed description of methylation as one of the major aging-related biochemical processes is provided in section 2.6). These changes may occur upon aging and/or exposure to environmental factors; they can be also inherited. They can also be called epimutations.^74^ The enzymatic systems ensuring the generation and maintenance of epigenetic alterations include the enzymes DNA methyltransferases, histone acetylases, deacetylases, methylases, and demethylases. They also include proteins involved in chromatin remodeling.^23,75^ Epigenetic alterations are profoundly involved in the process of aging, resulting in disturbances in the wide-ranging genome architecture, thus understanding the epigenome holds promise for amending age-related pathologies and prolonging healthy lifespan.^76,77^ In addition to age-related epigenomic changes, many other systems become dysfunctional with age.^23,31,33^ Noteworthy, chromatin regulation and transcription regulation have been identified to play major roles in the age-associated symptoms of these aging hallmarks.^78^ Given the reversible nature of epigenetic pathways, their understanding provides a promising approach for therapeutics against age-related decline and disease.^79^

As mentioned above, epigenetic alterations take place when methyl groups are added to or removed from a cytosine base (C) of DNA or when modifications are made to proteins called histones that bind to the DNA in chromosomes. DNA methylation produces DNA condensation, the form in which genes are not being transcribed. DNA methylation in mammals primarily occurs on the C5 of the cytosine base (5 methylcytosine, 5-mC) of CpG dinucleotides.^80^ Nearly 70–80% of CpG dinucleotides are methylated in somatic cells. Methylation levels change throughout life but generally tend to decrease upon aging. Global 5-methylcytosine (5mC) variations have been first described during aging in rats.^81^ DNA methylation affects a wide range of developmental and pathological processes. Further on, vast literature has documented genome-wide DNA methylation changes that occur in response to aging across multiple species.^82^

Although aging is largely correlated to changes in DNA methylation, the relationship between DNA methylation and aging is complicated. The general trends involve large-scale hypomethylation (non-CpG islands) and regions of hypermethylation (primarily CpG islands) upon aging.^83,84^ It is currently believed that DNA methylation biomarkers can verify biological age throughout the entire human lifespan. This phenomenon, known as an epigenetic clock, is based on CpG sites (cytosine and guanine bases separated by only one phosphate group in the DNA sequence) associated with age and the methylation profile of which can be used as an accurate indicator of biological age.^85−87^ Moreover, DNA methylation-based clocks are suggested as biomarkers of early disease risk and as forecasters of life expectancy and mortality.^82,88^ Thus, the Horvath clock defines a pattern of DNA methylation changes and considers the global decline in genomic CpG methylation as a well-documented predominant event in aging.^87,89,90^ A strong causative link between DNA and H3K9 methylation and aging is considered likely;^91−93^ however, the mechanisms underlying age-related DNA methylation alterations and aging mechanism are yet to be fully understood.

Histones are a family of basic proteins that provide structural support for the chromosomes. DNA winds around them to form nucleosomes, which are then wrapped around the chromatin fibers. In addition to their role in compacting genomic DNA into nuclei, histones also perform a structural function in regulating gene expression.^73^ An important feature of histone biology is their ability to acquire a large set of post-translational modifications that modulate their interaction with DNA or chromatin-associated proteins. Especially, the H3 and H4 histones, which exhibit long tails protruding from the nucleosome, can be covalently modified at several places (Table 1). These modifications have been reported to be important in gene expression profiles.^94^ Histone modifications also affect transcriptional accuracy; it is therefore conceivable that the observed loss in transcriptional precision with aging is causally related to histone modifications.^95^ The major modifications that impact them are methylation and acetylation (addition of an acetyl chemical group −COCH_3_). Methylation abnormalities result in enhanced cancer relapse with low survival rate. Alterations in methylation upon aging are accompanied by a loss in the acetylation level of certain histones (hypoacetylation). Animal model studies have reported that prevention of age-related hypoacetylation inhibits cognitive impairment and moderates illnesses such as Parkinson’s disease, osteoporosis, and stroke.^24,96^

**Table 1:** Age-Associated Changes in Histone Methylation

organism	histone modification	change
Mammals (mouse, rat, macaque)^97−100^	H4K20me3	↑
	H3K27me3	↑
	H3K79me1/2	↑
	H3K4me2	↑
	H4K20me1	↓
	H3K36me3	↓
Humans (HGPS^a^)^97,101,102^	H4K20me3	↑
	H3K9me3	↓
	H3K27me3	↓

a HGPS, Hutchinson–Guilford progeria syndrome.

Alterations in histone methylation have been considered as another characteristic feature of aging. In cases in which histone methylation has been found to impact aging, it does so by regulating transcription, categorizing it as a major mechanism of its action. Moreover, histone methylation regulates or is regulated by additional cellular pathways that contribute to or prevent aging.^97^ Cells from aged organisms show a large-scale loss of histones, specifically a gradual loss of histone H3 trimethylation at lysines 9 and 27 (H3K9me3 and H3K27me3), which are considered repressive marks that promote chromatin compaction. Another trend is the increase in “activating” histone marks (H3K4me2/3, H3K36me3).^23,24,97^ Some of these trends are exemplified in Table 1. Furthermore, it is worth noting that the role of histone methylation in aging is only starting to be appreciated.

Histone acetylation is a foremost regulator of transcription. It is known to promote transcription by reducing electrostatic interactions between DNA and histones and between neighboring nucleosomes.^103^ Histone acetylation is largely believed to function mostly via the cumulative charge effects of multiple acetylation event.^104−106^ Recent studies suggest limited selectivity of acetylation-directed antibodies; thus, most acetylation antibodies exhibit a polyacetylation bias and most H3 and H4 acetylation antibodies only barely discriminate among single individual acetylation events.^95,107^

Chromatin is a macromolecular complex of DNA and histone proteins that forms chromosomes within the nucleus of eukaryotic cells. It is a dynamic structure existing as either a compact and transcriptionally inactive heterochromatin or a decondensed transcriptionally active euchromatin (Figure 3B).^108,109^ Chromatin remodeling refers to the reorganization of chromatin from a condensed state to a transcriptionally accessible state, permitting transcription factors or other DNA binding proteins to access DNA and manage gene expression.^110^ The chromatin structure is reorganized by means of several mechanisms, including histone modification, histone tail separation, and ATP-dependent chromatin remodeling.^111,112^ The chromatin status can be modulated by environmental factors, which further modify the expression of genes related to aging and longevity.^113,114^

Noncoding RNAs, including long noncoding RNAs (lncRNAs), microRNAs (miRNAs), and circular RNAs, are important regulators of transcriptional networks and chromatin states; they have appeared as epigenetic factors that affect aging. Thus, lncRNAs stimulate gene silencing through interactions with chromatin-modulating enzymes and are emerging as important factors in the progression of aging.^115,116^ Noncoding RNAs are able to modify healthspan and lifespan by post-transcriptional regulation of stem cell behavior.^117^ Overall, studies suggest that RNAs may inherently impact aging and aging-related pathologies and represent likely therapeutic targets for deferring or ameliorating these pathologies.

Epigenomic changes, including modifications in transcription factors, histone features, nucleosome placement, and DNA methylation, are interrelated with the other hallmarks of aging.^23,118,119^ Epigenomic changes are able to activate the emergence of other hallmarks of aging and can also be influenced by them.^78^

#### 2.1.4 Disorders in RNA Processing

Robust alterations in expression pathways with advancing age have been reported, with a great part of these pathways controlling mRNA splicing. Furthermore, interventions that reverse senescent phenotypes help in restoring youthful patterns of splicing factor expression.^120^ It is believed that alterations in RNA processing add an additional level of gene expression regulation over those of genome integrity, transcriptional efficiency, and epigenetic regulation that have been already recognized to change during aging. Thus, dysregulation of RNA management regulation in aging human population has been identified as a characteristic aging feature.^121^

### 2.2 Loss of Protein and Organellar Turnover

#### 2.2.1 Loss of Proteostasis

Proteostasis denotes protein homeostasis, which includes the maintenance of stable functional proteins. Upon aging, proteostasis weakens. Aging cells accumulate misfolded and impaired proteins as a result of the functional decay in their protein homeostasis (proteostasis) mechanism, causing a lowered cellular viability and the development of protein misfolding diseases generically known as proteinopathies or protein conformational diseases, such as Alzheimer’s and Huntington’s diseases.^122^

The main participants in proteostasis preservation are the chaperones, the ubiquitin proteasome, and the lysosome-autophagy proteolytic systems. They take care of misfolded proteins, whether being refolded into the original stable conformation or being eradicated from the cell through proteolysis.^123,124^ Chaperones help *de novo* synthesized proteins and unfolded proteins to achieve their stable folded status. If folding happens to be unachievable, chaperones target the unfolded or misfolded protein for degradation by the proteasome or in lysosomes. The elimination of the misfolded proteins from the cytosol takes place by either degradation in lysosomes through autophagy or expulsion outside the cell by means of exosomes.^124^ All of these systems function in a synchronized way to restore the structure of misfolded proteins or to remove and degrade them entirely, thus preventing the accumulation of damaged materials. However, multiple studies have revealed that proteostasis is changed with aging.^125^

Chaperones accompany and safeguard proteins through each of their conformational changes including *de novo* folding, assembly and disassembly, transport through membranes, and targeting for degradation.^126^ Once targeting for degradation takes place, chaperones may decide which proteolytic pathway the misfolded protein will follow: through the proteasome (a multisubunit protease accountable for the degradation of proteins often tagged with ubiquitin) or through autophagy in the lysosomes. Certain age-related cellular alterations can influence chaperoning activities. The loss of chaperone function and a decline in their availability further worsen the difficulties with protein quality control. Improper age-related modifications in the substrate protein can also obstruct the chaperone’s capability to recognize its target. For example, accumulation of advanced glycation end-products via nonenzymatic alterations on long-lived proteins upon aging disturbs the normal chaperone activity (Table 2).^124^ Glycation, a spontaneous nonenzymatic reaction resulting in the formation of Amadori products, is one of the main biochemical processes that cause cellular damage and age-related diseases (a detailed description of glycation as one of the major aging-related biochemical processes is provided in section 2.6).

**Table 2:** Proteostasis System Mutations and Associated Age-Related Diseases^124^

proteostasis system mutations	age-related diseases
Chaperone mutations	
α-Crystallin	Early cataracts, desmin-associated myopathy, cardiomyopathy
DNAJB6	Hereditary myopathy
HSC70	Cardiovascular disease
HSJ1	Motor neuropathy (distal hereditary, dHMN)
HSP22, HSP27	Charcot–Marie–Tooth disorder
Sacsin	Spastic ataxia
Ubiquitin-proteasome system mutations	
Ataxin-3	Machado–Joseph disorder
PSMB8	Nakajo–Nishimura syndrome
Ubiquilin-2	Amyotrophic lateral sclerosis (ALS)
UCHL1	Parkinson’s disease
VCP/p97 (ERAD)	Paget’s disease, frontotemporal dementia
Autophagy system mutations	
ATG16L1	Crohn’s disease
LAMP2A	Cardiovascular disease, myopathy
p62	ALS, Paget’s disease
Parkin, PINK1 (mitophagy)	Parkinson’s disease
Presenilin-1	Familial Alzheimer’s disease

Proteasome activity and autophagy have also been reported to decline with aging.^127,128^ Stimulating proteasome or autophagy activity by overexpressing proteasome subunits or essential autophagy genes enhances lifespan and imparts resistance to stress in *S. cerevisiae*, *C. elegans*, and *D. melanogaster*.^129,130^ Evidence of such interventions in mammals is also emerging.^124^ Examples exist showing that genetic manipulations can improve proteostasis and delay aging in mammals.^131^

#### 2.2.2 Impaired Autophagy

Autophagy is a fundamental intracellular catabolic process used by cells to degrade and recycle components through lysosomes to balance their sources of energy and building blocks in an effort to maintain cellular homeostasis, differentiation, development, and survival upon stress.^33,132−134^ It involves the sequestration and transport of macromolecules and subcellular elements such as nucleic acids, proteins, lipids, and organelles to lysosomes for subsequent degradation.^135^ A major regulatory incident in autophagy instigation is exerted by the initiation complex interactions with the nutrient-sensing mTOR kinase and the energy-sensing AMP-activated protein kinase (AMPK), both of which are recognized inducers of autophagy in response to stress. Thus, autophagy initiation is controlled by both nutrient- and energy-sensing mechanisms.^136^

A growing body of evidence indicates that autophagy activity deteriorates with age in various organisms.^137^ Upon aging and neurodegeneration, flaws in certain steps of autophagy regulation have been observed, which result in the accumulation of damaged organelles and protein aggregates. They are harmful for cell metabolism and homeostasis, which further worsens imperfect autophagy.^134,138^ Noteworthy, activation of autophagy has been reported to increase mouse lifespan^139^ and even enhance immune response to vaccination in older individuals by defeating immunosenescence.^140^ Autophagy is in close correlation with numerous other hallmarks of aging and is currently considered as an integrative aging feature.^141^ It is critical for maintaining protein homeostasis (proteostasis). Autophagy work together with the ubiquitin proteasome system to destroy toxic proteins.^124^ Upon aging, postmitotic cells exhibit compromised proteostasis, which correlates with the functional decay of protein quality control tools, including autophagy.^124^

Autophagy plays a well-documented role in eliminating dysfunctional mitochondria, termed mitophagy.^142^ It has been also demonstrated that autophagy controls cellular senescence, the process of steady proliferation suppression of mitotic cells initiated by diverse stresses, including telomere attrition, DNA impairment, mitochondrial dysfunction, and abnormal hyperproliferative stimuli.^143^ Altogether, loss of autophagy generates various cellular malfunctions that exacerbate aging.^137^ Since reduced autophagy is implicated in multiple age-related diseases, including neurodegeneration, sarcopenia, and osteoarthritis, therapeutic autophagy upregulation has potential toward treating such age-related disorders.

### 2.3 Metabolic Alterations

Metabolic modifications are the most common symptoms of aging in cells. They include deregulated nutrient sensing and mitochondrial dysfunction as well as accumulation of sphingolipids and dysregulation of cholesterol metabolism. Such changes increase the risk of age-associated diseases, such as type 2 diabetes, stroke, and hypertension. Insulin resistance is the foremost metabolic syndrome noticed in older adults.

#### 2.3.1 Deregulated Nutrient Sensing

Nutrients are substances needed by the body to sustain basic functions in order to survive, grow, and reproduce and are optimally obtained by eating a balanced diet. Thus, glucose and other carbohydrates, amino acids, and lipids are essential cellular nutrients with certain mechanisms to sense their availability in mammalian cells. The capability to sense and respond to variations in the environmental nutrient availability is a key requisite for survival. Thus, cells must be able to store nutrients when they are abundant and access them when they are scarce. Moreover, nutrient levels in the circulation need to stay within certain safe ranges. Therefore, cells must be able to sense nutrient levels in order to react appropriately. Various pathways that sense intracellular and extracellular levels of carbohydrates, amino acids, lipids, and different metabolites are integrated and coordinated at the organismal level via hormonal signals. Throughout food abundance, nutrient-sensing pathways employ anabolism and storage, whereas food scarcity activates homeostatic mechanisms.^144^

There are four nutrient sensing pathways:

- Insulin and IGF-1 signaling (IIS)
- Mechanistic target of rapamycin (mTOR)
- AMP-activated protein kinase (AMPk)
- Sirtuins

The IIS and mTOR pathways indicate nutrient abundance, so downregulating them prolongs the lifespan by reducing cell growth and anabolic metabolism. On the other hand, the AMPk and sirtuin pathways imply nutrient scarcity, so their upregulation prolongs lifespan by reducing nutrient sensing, thus imitating dietary restriction. Some adverse effects caused by upregulating or deregulating these nutrient signaling pathways include compromised wound healing, insulin resistance, cataract formation, and testicular degeneration upon mTOR pathway downregulation by rapamycin administration.^145^ Nutritional antiaging strategy known as calorie restriction has been successfully examined in diverse eukaryotic species.^146^ Research efforts have been focused on outlining the molecular mechanisms linking metabolic balance induced by calorie restriction and the biology of aging, thus revealing the key significance of nutrient sensing upon aging.^147^

Amino acids regulate multiple interacting nutrient sensing pathways. The adequate sensing of amino acid availability is significant for the effective regulation of protein synthesis and catabolism. An important way of amino acid control for nutrient sensing is via the amino acid sensing taste receptors.^144^ Taste receptors are members of the T1R and T2R families of G-protein-coupled receptors. Amino acid taste receptors in humans exhibit a high affinity to glutamate, yet other l-amino acids also operate as ligands, while d-amino acids do not.^148^ In a similar way to amino acid taste sensing by T1R1–T1R3, a T1R2–T1R3 heterodimer constitutes the glucose taste receptor, which is activated by millimolar concentration of glucose, fructose, or sucrose.^149^

Deregulated nutrient sensing ability takes place upon aging.^150^ The significance of nutrient sensing throughout the aging process has been first established in the prominent observation that decreased food intake in rats prolongs lifespan relative to *ad libitum* fed controls.^151^

One of the predominant nutrient sensing dysfunctions that occur upon human aging is insulin resistance. Upon aging, factors including oxidative stress, inflammation, enzymatic activity disorders, and fatty acids accumulation in cells can all contribute to a decline in insulin sensitivity. These changes can be driven by many other aging denominators. As a consequence, the body gradually loses its capability to regulate blood sugar level, with the pancreas producing more insulin in an effort to compensate. Insulin resistance increases inflammation and oxidative damage, promotes glycation, and alters fat metabolism in liver, thereby advancing atherosclerosis and fatty liver disease.^152^

#### 2.3.2 Mitochondrial Dysfunction

Mitochondria are rightly known as the cell’s powerhouses, converting nutrients into energy that can be used by the cell. Mitochondrial damage impairs the ability to fuel the cell. The main source of such damage is free radicals, natural byproducts of energy production in the mitochondria.

Reactive oxygen species (ROS) is a group of species that includes hydrogen peroxide (H_2_O_2_), superoxide ion (O_2_^•–^), and hydroxyl radical (^•^OH). ROS are highly reactive species that have been believed to be the primary source of endogenous oxidative stress damage. They are outcomes of the oxidative metabolism in mitochondria, typically scavenged by the superoxide dismutase (SOD) enzyme (a detailed description of oxidation as one of the major aging-related biochemical processes is provided in section 2.6). Upon mitochondrial malfunction, ROS are released producing oxidative damage to mitochondrial and cellular DNA.^153−156^ These reactions signal a DNA damage response similar to that produced by telomere shortening, causing senescence.^155^ Thus, changes in mitochondrial biology resulting in enhanced ROS concurrently alter the epigenetic status at the DNA methylation level. Moreover, DNA methylation and histone acetylation vary upon aging and convey modifications in expression of mitochondrial genes, thus producing a feedback loop of failing mitochondrial function.^153,157^

Senescent cells undergo significant changes in their mitochondrial function, dynamics, and morphology.^158^ They exhibit decreased membrane potential, higher proton leak, intensified enzyme release, higher mass, and higher amount of tricarboxylic acid cycle metabolites.^159^ The number of mitochondria in senescent cells is enhanced, due to the accumulation of old and dysfunctional mitochondria because of deficient mitophagy (mitochondrial removal).^160^ Furthermore, mitophagy deficiency appears as a distinct mechanism for mitochondrial mass expansion.^158,161^

Regardless of their abundance, mitochondria in senescent cells typically exhibit a lower ability to produce ATP.^162^ Instead, senescent cells are characterized by a Warburg shift (a shift from oxidative phosphorylation to rapid aerobic glycolysis); this produces more ROS, thus causing protein and lipid damage, telomere shortening, and DNA damage response.^157,158,163^

#### 2.3.3 Accumulation of Sphingolipids and Dysregulation of Cholesterol Metabolism

Recent data point to a new mechanism of aging: the accumulation of sphingolipids.^34,35^ Ceramides, a common class of sphingolipids, build up in aging muscles, driving down their function and affecting the functional ability of older adults. Thus, it has been reported that inhibiting ceramide production in cells could prevent sarcopenia or muscle loss associated with aging. Administration of myriocin (a drug shown to inhibit the production of ceramides) to aging mice slowed sarcopenia, maintaining their muscle strength. It has been reported that the effects were related to muscle stem cell operation; when ceramide production has been inhibited, the number of muscle stem cells and their operational ability have been better preserved.^34,36^ The study opens up a new research approach regarding the effect of ceramides on aging and stimulates the development of prospective therapeutic strategies involving sphingolipids in humans.

### 2.4 Cellular Senescence, Stem Cell Exhaustion and Altered Intercellular Communication

Cellular senescence, stem cell exhaustion, and altered intercellular communications are aging attributes that have an effect mainly at the cellular level.

#### 2.4.1 Cellular Senescence

Cellular senescence denotes cells that have entered a status of arrested growth in reaction to cellular damage (Figure 3C). Thus, senescent cells lose productiveness and no longer divide; they also trigger growth in inflammation, which can aggravate aging.^153^ Even though all cell types are able to undergo senescence upon aging, it mainly impacts fibroblasts, endothelial cells, and immune cells.^164,165^ Even postmitotic or slowly proliferating cells, such as the brain or the heart cells, may experience senescence.^166^ Senescent cells exhibit modifications in their metabolic activity, undergo significant changes in gene expression, and develop a complex senescence-associated secretory phenotype (SASP), composed of proinflammatory cytokines, chemokines, growth factors, and matrix-remodeling enzymes that are able to alter their microenvironment.^167,168^ Cellular senescence can impair tissue repair and regeneration, thereby promoting aging. Cellular senescence has been associated with multiple age-initiated disorders, such as cancer, diabetes, osteoporosis, cardiovascular disorders, stroke, Alzheimer’s disease, and dementias, as well as osteoarthritis.^169^ It has also been related to declines in the eyesight, mobility, and cognitive capability.

It has been reported that continuous removal of senescent cells by genetic or pharmacological interventions extends the longevity and health of aged mice, verifying the key role of cellular senescence in aging.^170^ Thus, removal of senescent cells can attenuate age-related tissue dysfunction and extend the health span.

Senescence can be triggered by different types of stress. Cells can go through senescence in response to various stimuli, such as telomere shortening, alterations in telomeric structure, mitogenic indications, oncogenic stimulation, radiation, oxidative stress, epigenetic alterations, chromatin disorders, loss of proteostasis, mitochondrial dysfunction, inflammation, tissue damage, and nutrient deficiency.^171−176^

Senescence can also perform as an effective antitumor mechanism, by inhibiting proliferation of cancer cells during carcinogenesis.^177,178^ It is a cellular framework that exhibits both favorable and harmful effects on the health of an organism, a supposed instance of evolutionary antagonistic pleiotropy. Initiation of the p53/p21^WAF1/CIP1^ and p16^INK4A^/pRB tumor suppressing pathways, which is actuated in response to DNA damage produced by telomere attrition and oxidative or oncogenic stress, performs a key role in controlling senescence. Several other pathways have recently been associated with mediating senescence and the senescent phenotype.^179^ Better in-depth knowledge of the mechanisms regulating senescence may provide promising translational prospects to develop novel therapeutic strategies that minimize the harmful consequences of senescence. Targeting senescence by senolytic drugs to selectively eradicate senescent cells or control SASP using small molecules or antibodies will facilitate treatment of senescence related disorders and may contribute toward expanding healthspan.

#### 2.4.2 Stem Cell Exhaustion

Stem cells play a critical role in tissue repair and regeneration.^145^ However, as an organism ages, its function declines, leading to a reduction in tissue regeneration capacity and an increased risk of age-related diseases. Stem-cell exhaustion indicates stem cells and progenitor cells accruing damage over time and eventually becoming depleted upon aging. Thus, aging is accompanied by a continuous decrease in tissue renewal, as well as by compromised tissue repair upon injury.^180,181^

This decline in stem cell function is due to a variety of factors, including increased cellular damage, changes in gene expression, and alterations in the microenvironment surrounding the stem cells. The suggested mechanisms of stem cell exhaustion include the following:

- Telomere shortening
- DNA damage accumulated upon aging caused by a variety of factors, including oxidative stress, radiation, and chemical exposure
- Epigenetic modifications, such as changes in DNA methylation or histone modifications, can alter gene expression and affect stem cell function. These changes can accumulate over time and contribute to stem cell exhaustion.
- Alterations in the stem cell microenvironment. The microenvironment, or niche, surrounding stem cells is critical for their function. Age-related changes in the niche, such as decreased nutrient and oxygen supply or the accumulation of toxic metabolites, can impair stem cell function.^181−183^

Studies have shown that stem cell exhaustion is a major contributing factor to age-related declines in tissue regeneration, including the loss of muscle mass, impaired bone healing, and decreased skin elasticity. Stem cell exhaustion also increases the risk of age-associated disorders such as Alzheimer’s disease, cardiovascular disease, and cancer. Tissue repair is supposed to largely rely on injury-induced cellular dedifferentiation and plasticity. Thus, in certain tissues, injury induces dedifferentiation of multiple non-stem-cells acquiring stem cell properties, attaining the plasticity necessary for tissue repair.^184^

There is ongoing research to investigate strategies to reverse stem cell exhaustion and restore their regenerative capacity. These strategies include genetic manipulation, cellular reprogramming, and the use of growth factors and other compounds that stimulate stem cell proliferation and differentiation.^185^

#### 2.4.3 Altered Intercellular Communication

Aging causes modifications in cell signaling at every level. Neuronal and hormonal signaling gets deregulated, causing enhanced inflammation (inflammaging), reduced immune performance (immunosenescence), and alterations in the extracellular surroundings.^153^ Altered intercellular communication implicates the change in signaling between cells, possibly leading to certain diseases and disabilities of aging. The age-dependent alterations in intercellular communication integrate the effects of other features of aging. Specifically, senescent cells initiate chronic inflammation, which can further damage aging tissues. Thus, multiple factors bring about the altered intercellular communication, one of which (the SASP) is directly triggered by the cellular senescence.

In addition to the above well-recognized hallmarks of aging, recently more distinctive features of that process have been identified.^31,33,34,36,186^

#### 2.4.4 Microbiome Disorders: Dysbiosis

The importance of gut microbiome in many aspects of human health is currently well recognized.^187^ Recent progress in next generation sequencing tools has made possible the identification of prominent changes in the gut microbiome upon aging, indicating specifically certain shifts in microbial populations and loss of species diversity.^188^ Such an imbalance in the gut microbial community is referred to as dysbiosis. Along with age-related deficiency of structural integrity of the gut and other physiological barriers, such shift in microbial populations can trigger inflammation and other disorders.^189,190^

Age-associated changes in the gut microbiota include a decrease in microbial diversity, an increase in the abundance of potentially harmful bacteria, and a decrease in the abundance of beneficial bacteria. In particular, there is often an increase in the abundance of potentially pathogenic bacteria, such as Proteobacteria, and a decrease in the abundance of beneficial bacteria, such as Bifidobacteria.^191^ These changes can contribute to a variety of health issues that are more common in older adults, such as constipation, inflammation, and impaired immune function.

Aging is also associated with changes in the structure and function of the intestinal barrier, which can lead to increased intestinal permeability as well as the translocation of bacteria and bacterial products into the systemic circulation. This can result in low-grade inflammation and immune activation, which are supposed to contribute to the development of age-related diseases.^192,193^

Aging can also cause impaired immune function, including a decline in the function of innate immune cells such as macrophages as well as a decrease in the diversity and function of T and B cells. These changes can lead to impaired immune surveillance of the gut microbiota and the decreased ability to respond to pathogens.^194,195^

Dysbiosis has been linked to a variety of age-related diseases including metabolic disorders, cardiovascular disease, cognitive decline, and frailty. However, it is not yet clear whether dysbiosis is a cause or consequence of these conditions. There is growing interest in developing interventions to promote healthy gut microbiota in older adults, with the goal of preventing or mitigating the effects of age-related dysbiosis. Potential interventions include prebiotics and probiotics, dietary interventions, fecal microbiota transplantation, and even microbial therapeutics, such as bacteriophages. Maintaining a healthy gut microbiota through healthy lifestyle habits and interventions may help to promote healthy aging.^194−197^

#### 2.4.5 Chronic Inflammation: Inflammaging

Chronic inflammation, also known as “inflammaging”, is a low-grade, persistent, and systemic state of inflammation that occurs upon aging and is currently considered a key biological basis of the aging process.^198−200^ It is believed to be caused by the accumulation of cellular damage and the failure of the immune system to clear damaged cells efficiently. This results in the release of certain inflammatory mediators in the blood, including IL-1, IL-6, C-reactive protein, and IFNα.^200^ Chronic inflammation has been linked to a wide range of age-related diseases, including cancer, diabetes, cardiovascular disease, and neurodegenerative diseases, as well as atherosclerosis, neuroinflammation, osteoarthritis, and intervertebral disc degeneration.^201^ It is also associated with a decline in physical and cognitive function as well as an increased risk of disability and mortality.

While the exact mechanisms behind inflammaging are not fully understood, researchers believe that a variety of factors can contribute to its development, including lifestyle choices such as poor diet, sedentary behavior, and smoking, as well as environmental exposures such as pollution and toxins.^202^

Inflammaging is related to other characteristic features of aging process such as cellular senescence and the disturbances in gut microbiota known as dysbiosis.^203^ It might be triggered by ineffective/disabled autophagy and genomic instability.^31^ Overexpression of proinflammatory mediators can be a result of epigenetic dysregulation or deficient proteostasis.^186^ Inflammaging is aggravated by disturbances of the circadian rhythm as well as by gut barrier dysfunction.^204^ A recent study correlated mitochondrial dysfunction with inflammaging implying that reduced mitochondrial calcium uptake in macrophages seems to be a major driver of age-associated inflammation.^205^

Reducing chronic inflammation may be an important strategy for improving health and preventing age-related diseases. Lifestyle interventions such as regular exercise, healthy diet, stress reduction, and adequate sleep have been shown to reduce inflammation and improve health outcomes.^206^ Additionally, certain medications and supplements may also be effective at reducing inflammation. However, more research is needed to fully understand the complex mechanisms behind inflammaging and to develop effective interventions, reducing its impact on aging.

#### 2.4.6 Mechanical Properties Alterations

Cellular and extracellular mechanical property alterations take place upon aging. Fibroblast senescence is associated with a change in actin, from a f-actin that can be polymerized and depolymerized upon cell motility, to f-actin fibers, which are likely to impact cell motility and cell–cell communication.^207^ Motility changes are of significant importance for the innate immune system aging, in which neutrophils from aging individuals induce substantial tissue damage upon migration to sites of inflammatory signaling.^208^ The nucleoskeleton also undergoes changes upon aging, with the nuclear lamina becoming destabilized and concomitant extrusion of chromatin into the cytoplasm, triggering the SASP in senescence.^209^ Lastly, the extracellular matrix also changes with aging, which greatly affects cell performance.^210^ Enhanced rigidity and loss of elasticity, as a result of glycation cross-linking between collagen molecules, can be in charge of multiple age-related disease conditions such as hypertension with related kidney and neurological disorders (a detailed description of glycation as one of the major aging-related biochemical processes is provided in section 2.6). The field of biomechanics is thus considered highly relevant to the physiology aging and antiaging strategies.^33^

### 2.5 Hallmarks of Aging Are Interrelated

**Figure 4:**
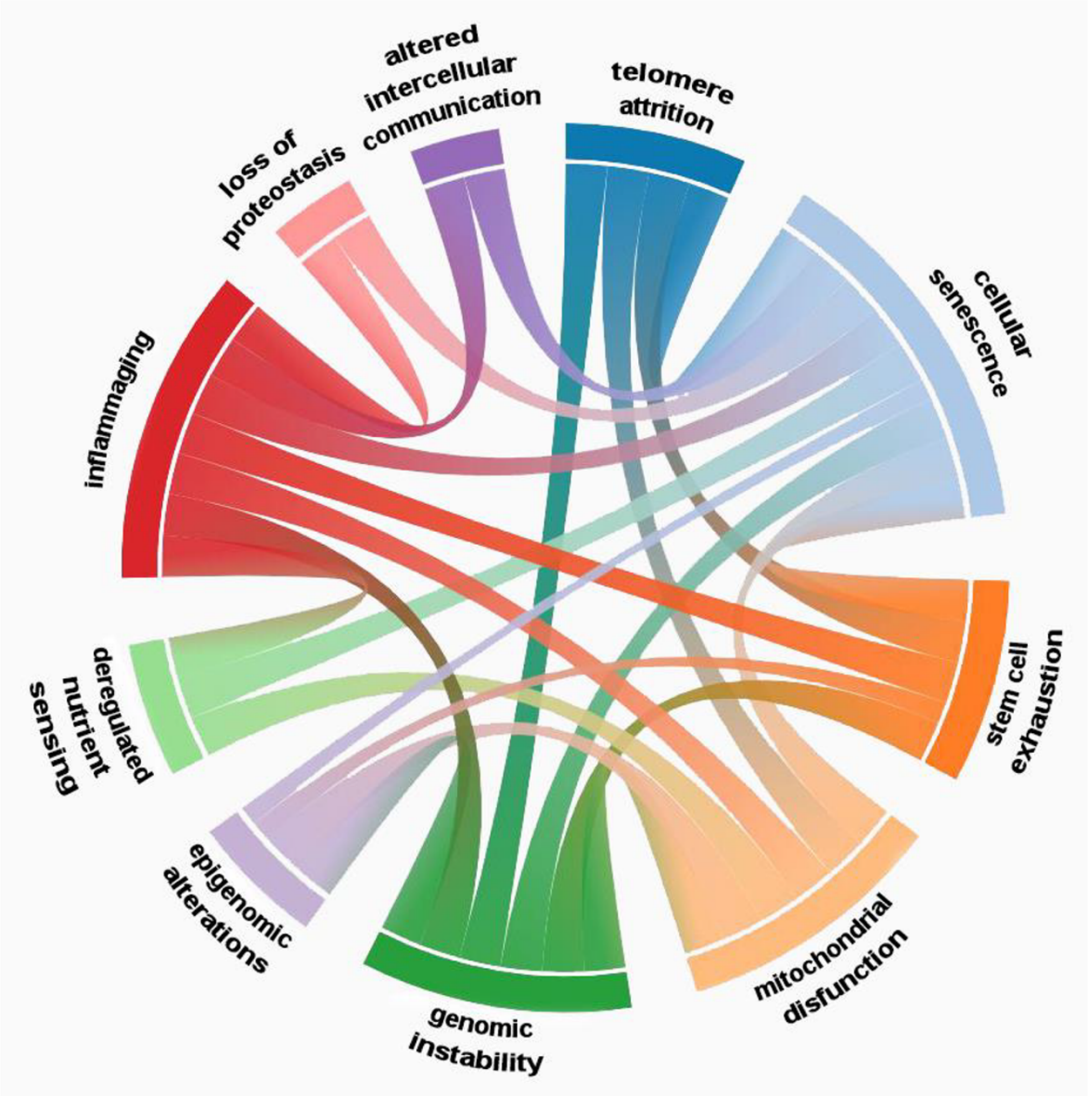
Interrelations between the hallmarks of aging.

Overall, the various hallmarks of aging are interconnected and can contribute to each other (Figure 4).^23,118,119^ For example, cellular senescence can promote inflammation, which can further exacerbate mitochondrial dysfunction and genomic instability. Similarly, genomic instability can lead to epigenetic alterations, which can impact the function of stem cells and contribute to their exhaustion.^23,30,31^

- Genomic instability can also contribute to telomere attrition and cellular senescence. Conversely, telomere attrition can also contribute to genomic instability, as shortened telomeres can lead to DNA damage and mutations. Epigenetic alterations can impact genomic instability and cellular senescence. Loss of proteostasis, deregulated nutrient sensing, mitochondrial dysfunction, altered intercellular communication, and stem cell exhaustion can all contribute to cellular senescence and inflammation. Cellular senescence can impact genomic instability and telomere attrition.^211−213^
- Epigenetic alterations can affect gene expression, including the expression of genes that regulate cell growth and senescence.^214,215^ For example, certain epigenetic changes can lead to the upregulation of p16 and p21, two proteins that promote cellular senescence.^216^
- One of the consequences of loss of proteostasis is the accumulation of misfolded and damaged proteins. This can trigger cellular senescence, as cells can activate senescence pathways in response to protein stress.^217^
- One of the pathways that regulate nutrient sensing is the mTOR pathway. Dysregulation of this pathway can lead to increased mitochondrial dysfunction, as mTOR can impact mitochondrial biogenesis and function.^218,219^
- Senescent cells can secrete a variety of molecules, including cytokines and growth factors, that can impact the function of neighboring cells. This can contribute to altered intercellular communication.^220^
- Mitochondrial dysfunction resulting in increased ROS, concurrently brings about epigenetic alterations at the DNA methylation level. DNA methylation and histone modifications upon aging impart modifications in gene expression of mitochondrial genes, creating a feedback loop of declining mitochondrial function.^157^

Understanding the relationships among the different hallmarks of aging can help in developing effective interventions to prevent or treat age-related diseases.

As an alternative approach to considering aging as a set of isolated processes in terms of discrete hallmarks, it has been suggested to consider aging as involving four layers, each at a different biological scale.^119^ From a general phenotype to a molecular mechanism, the suggested four layers of aging include (i) a decline in physical function of the organism and increased susceptibility to diseases; (ii) systemic immune, metabolic, and endocrine malfunction; (iii) cellular dysfunction; and (iv) failure of biomolecule performance.^119^ Failures within each layer and relations between them allegedly generate the aged phenotype and its associated susceptibility to diseases.

### 2.6 Major Biochemical Processes Related to Aging

The three main biochemical processes that cause cellular damage and age-related diseases include methylation, glycation, and oxidation.

#### 2.6.1 Glycation

**Figure 5:**
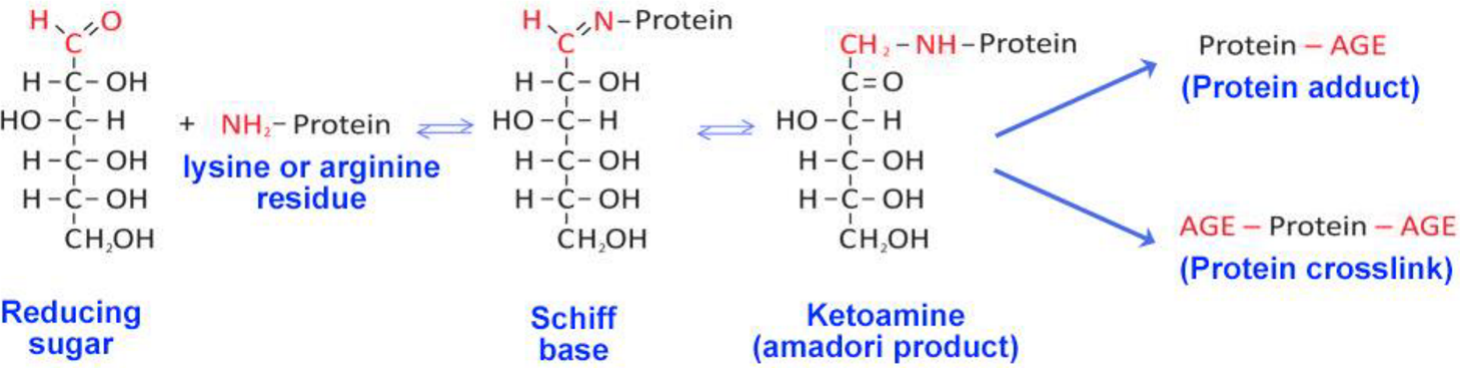
Scheme of the Maillard reaction. Reducing sugar reactive carbonyl groups react with the proteins amino groups to form a Schiff base, which further rearranges to more stable Amadori products. These early glycation end products further form either protein adducts or protein cross-links.

Glycation is a spontaneous nonenzymatic reaction of free reducing carbohydrates with free amino groups of proteins, nucleic acids, and lipids, which results in the formation of Amadori products (Figure 5).^221^ Further, these Amadori products go through an assortment of irreversible dehydration and reorganization reactions leading to the development of advanced glycation end products (AGEs).^222^ The glycation reaction leads to protein function deficit and reduced elasticity of biological tissues such as blood vessels, skin, and tendons.^223,224^ The glycation process is augmented in the presence of hyperglycemia and oxidative stress.^225^ Since there are no enzymes to eliminate glycated products from the organism, glycation complies with the theory that the accumulation of metabolic waste promotes aging. A set of exemplary advanced glycation end products (AGEs) are listed in Table 3, along with the number of related documents in the CAS Content Collection.

**Table 3:**
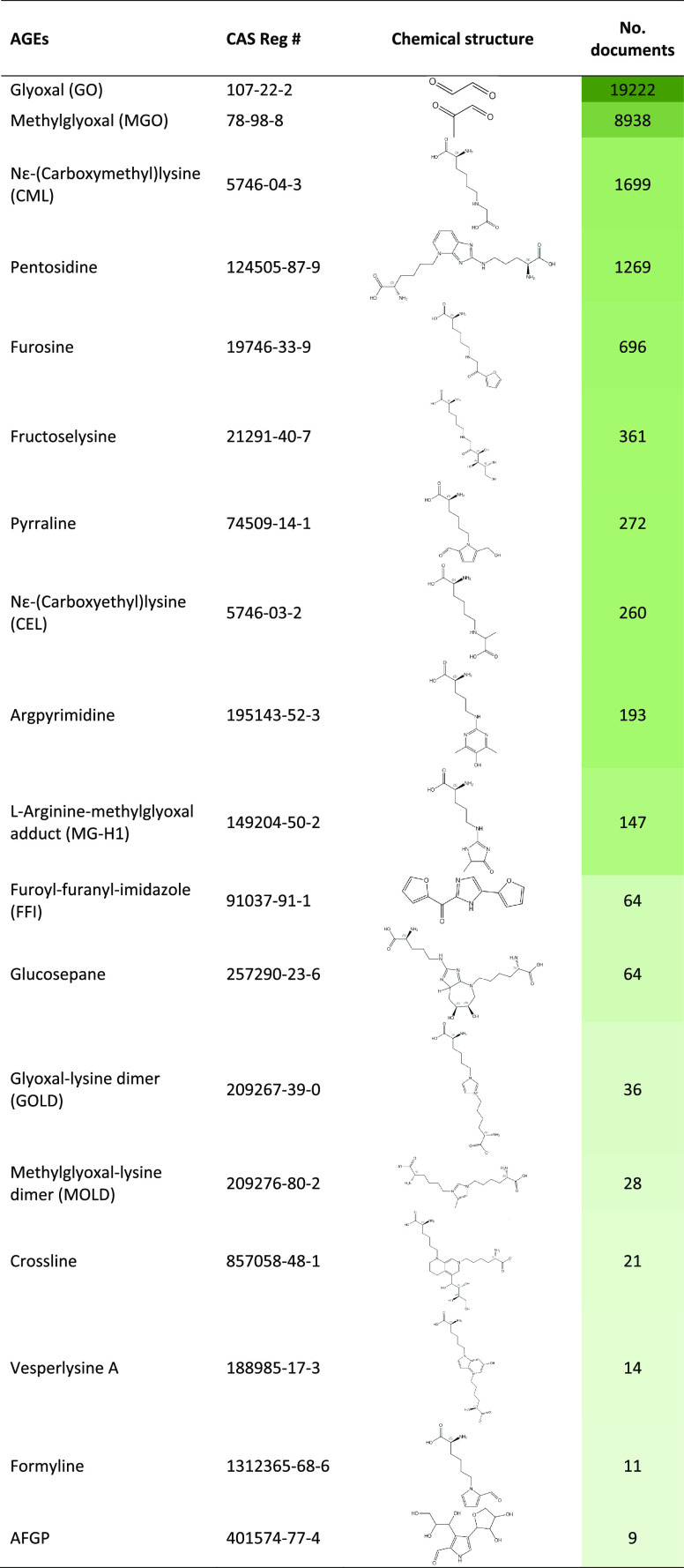
Chemical Structures of Exemplary Advanced Glycation End Products (AGEs) and the Number of Related Documents in the CAS Content Collection

#### 2.6.2 Oxidation

Oxidative stress has been assumed to notably contribute to aging.^226−230^ The oxidative stress theory of aging hypothesizes that age-related decline in physiological performance is caused by a slow continual accumulation of oxidative damage to biomolecules, which grows with age and is associated with life expectancy decline of organisms.^231^ Oxidative damage contributes to multiple hallmarks of aging and drives multiple age-related diseases. Thus, telomeres are highly sensitive to oxidative damage.^232,233^ Therefore, oxidative damage may cause telomere attrition, which accelerates aging and augments the risk of age-related diseases.^234^ Oxidative stress has been defined as an imbalance between the production of oxidants and their elimination by antioxidants, leading to disturbance of redox signaling and control, and/or molecular impairment.^228^

According to the oxidative stress theory of aging, damages caused by free radicals are the main reason of aging and a shorter lifespan.^235−237^ ROS are highly reactive species, mainly including free radicals comprising at least one unpaired electron (superoxide radicals (O_2_^–•^), hydroxyl radicals (^•^OH), and hydrogen peroxides (H_2_O_2_)), and have been believed to be the primary source of endogenous oxidative stress damage.^238^ It is widely agreed that the largest part of ROS are produced by the electron transport chains of mitochondria during regular oxidative respiration in addition to numerous intracellular pathways.^239^ The principal process of ROS production in mitochondria can be schematically described as O_2_ → O_2_^–•^ → H_2_O_2_ → ^•^OH.^239^

**Figure 6:**
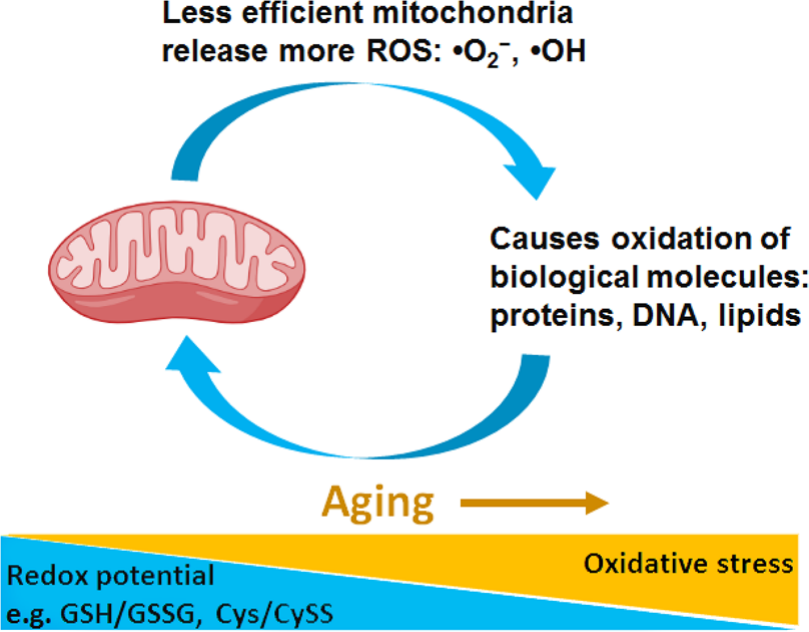
Schematic presentation of the positive feedback loop between mitochondria dysfunction and oxidative stress upon aging.

Furthermore, ROS are generally produced by mitochondria throughout physiological and/or pathological processes. Thus, O2^•–^ can be formed by cellular respiration, by lipoxygenases and cyclooxygenases via the arachidonic acid metabolic pathway, and by endothelial and inflammatory cells.^240^ In the electron transport chain, oxygen molecules have been reduced into O_2_^–•^ with a leak of electrons, with the formation of superoxide being the initial step in a cascade reaction of other ROS generation. When generated, it can be catalyzed by superoxide dismutase (SOD) into H_2_O_2_. Next, in the presence of reduced form transition cation (Fe^2+^ or Cu^+^, known as a Fenton reaction) or myeloid peroxide, H_2_O_2_ further converts to ^•^OH. Meanwhile, H_2_O_2_ can also be reduced into H_2_O by the enzymatic antioxidants such as catalase and glutathione peroxidase. Haptoglobin steadily binds hemoglobin with strong affinity, inhibiting the release of heme iron from hemolysis into systemic circulation, therefore terminating the Fenton reaction and avoiding the production of ^•^OH. Mitochondrial dysfunction upon aging results in increased ROS, thus causing enhanced oxidation of biomolecules (proteins, DBA, lipids) and opening a positive feedback loop of aging damage (Figure 6).

Noteworthy, examining the hypothesis of oxidative stress in aging and diseases has disclosed controversial results. There is a sizable evidence that macromolecular oxidative damage rises with age and seems to be related to life expectancy in multiple organisms. Yet, a direct relationship between oxidative damage and aging has not been conclusively established.^241^ In fact, the role of ROS in the body is complex, and its effects on health vary largely along with changing ROS levels. Within physiological levels, ROS facilitate the preservation of cellular homeostasis and performance.^228^ Therefore ROS levels can exhibit both favorable and detrimental effects, as suggested in the concept of mitohormesis.^242^

Oxidative stress may result in damage to various classes of biomolecules, including lipids, proteins, and nucleic acids. Polyunsaturated fatty acid (PUFA), especially those with a higher number of double bonds, are highly susceptible to lipid peroxidation by an autocatalytic oxidative chain reaction.^243^ Peroxidation of phospholipids in lipid membranes may result in a decline in membrane fluidity and permeability and thus inactivation of membrane receptors, resulting in cell apoptosis. Moreover, lipid radicals generated during oxidation can form a multitude of harmful end products, including reactive aldehydes, alkanes, and alkenes.^244^

Proteins are also key targets for ROS. Protein oxidation includes the following: (i) oxidative alteration of amino acid residues, particularly cysteine and methionine; (ii) fragmentation as a result from oxidative cleavage of the peptide backbone; (iii) production of protein carbonyl derivatives; (iv) protein cross-links generation.^245−247^ Protein oxidation may cause changes in their three-dimensional structures, alteration of their physiological features such as enzyme performances and signal transduction, and further proteolytic degradation/aggregation of proteins, partial unfolding and modified conformation.^246,248,249^

ROS, specifically the hydroxyl radicals, can incite oxidative damage to the nuclear DNA, including base mutation, strand breaking, DNA–protein cross-linking, and DNA-adducts formation.^239^ Overall, hydroxyl radicals can react with DNA bases and sugar–phosphate backbone, leading to inaccurate base pairing and further common mutations.^250^ Hydroxyl radicals can also react with the deoxyribose moiety, resulting in loss of DNA bases and DNA breaks. Such breaks are documented risk factors of genome instability, cell cycle disruption, and cell death.^251−253^ DNA–protein cross-links involving thymine and tyrosine in the nucleoprotein complex of histones and DNA can also be activated by the hydroxyl radicals.^254^

#### 2.6.3 Methylation

Recent research progress provides convincing evidence of genomewide DNA methylation changes upon aging and age-associated diseases. Methylation is a process in which a methyl group (−CH_3_) is attached to a cytosine base (C) of DNA. It initiates DNA condensation, a configuration in which genes have not been transcribed. Methylation levels change throughout life but generally tend to decrease upon aging.

The methylation reaction is catalyzed by DNA methyltransferases (DNMTs), enzymes transferring a methyl group from the *S*-adenosyl-l-methionine (SAM) to the C5 of a cytosine. Such a reaction includes SAM as an electrophile methyl donor and C5 as a weak nucleophile incapable of interacting with SAM by itself. However, a nucleophile from a DNMT can bind covalently to the carbon-6 of cytosine, which activates the nucleophilic nature of C5, enabling the transfer of a methyl group from SAM. The enzyme nucleophile is consequently removed and deprotonation at C5 breaks up the nucleotide–DNMT complex.^255^ DNA methylation typically leads to gene silencing.^256^ There are various routes to gene silencing through methylation. Since the greater part of mammalian transcription factors exhibit DNA recognition elements containing motifs rich in CpG, as well as GC-rich binding sites, DNA methylation can block or abolish their capability to act on many significant regulatory sites.^257^

Histone methylation is a reaction in which methyl groups are relocated to the amino acids of histone proteins. These proteins participate in the fundamental unit of chromatin, the nucleosome. The DNA double helix wraps around the nucleosomes to form chromosomes. Histone methylation is critical for the regulation of gene expression by controlling the chemical attractions between histone tails and DNA.^97,112^

The question of whether alterations in methylation are the result of aging and pathology or in fact one of its contributing factors has not been decisively solved yet. A wide variety of age-associated diseases exhibit abnormal methylation, and many prospective treatments based on rejuvenating the methylome are yet unexplored. Future research will require a better understanding of the alleged mechanisms surrounding DNMTs and their associated partners in DNA methylation. Advanced research into methylomic aging- associated diseases, drug discovery, and regulatory mechanisms is essential to uncovering the function of DNA methylation in aging, rejuvenation, and age-associated diseases.

### 2.7 Age-Related Diseases

Decline of bodily functions upon aging is a major risk factor for crucial human pathologies. Moreover, because advanced age is the common inherent cause, such chronic disorders frequently take place concurrently as comorbidities in the elderly population.^23,258−260^ Among these major pathologies are cancer and cardiovascular disorders. Age-associated diseases impacting the musculoskeletal system are common as well, particularly osteoarthritis, osteoporosis, and sarcopenia. Metabolic disorders such as diabetes and hepatic steatosis are also common with age. Organ and tissue fibrosis, a pathological progression typified by excessive fibrous connective tissue production,^261^ also raises upon aging and is one of the main causes for age-related deterioration of human organs. Overall weakening of the immune system increases susceptibility to infectious diseases.^262^ Neurodegenerative diseases, such as Alzheimer’s, Parkinson’s, and Huntington’s diseases, and sensorial malfunctions such as auditory and macular degeneration all increase considerably upon aging.^18,259,263,264^

- **Cardiovascular disease** is the most frequent cause of death in older adults. This disease class mainly includes coronary artery disease, congestive heart failure, and arrhythmia. Vascular stiffing and remodeling are known to take place throughout normal aging.^265,266^
- **Atherosclerosis** progresses as cholesterol, fat, and other substances in blood form plaques, which cause narrowing of the arteries. This decreases the supply of oxygen-enriched blood to tissues and organs in the body.^266^ Atherosclerosis triggers inflammation and further vascular changes, thus enhancing risk for cardiac and cerebrovascular disorders, peripheral vascular disease, cognitive impairment, and other cardiovascular damage.^265,267^
- **Cerebrovascular disease (stroke)** is another common age-related disease. Stroke happens when blood stops flowing in an area of the brain as a consequence of a disruption of a blood vessel. It is a very critical condition because brain cells deprived of oxygen die quickly, so it can cause death or serious disability.^268^
- **Hypertension**, the most common chronic disease of older adults, is the major promoter of atherosclerosis.^269^ However, the worth of intensive pharmacotherapy for hypertension in people over age of 75 remains controversial.^265^ Current belief is that aggressive treatment needs to be offered and continued as long as it is well-tolerated.^269^
- **Cancer** is the second leading cause of death in older adults, most commonly lung, breast, prostate, and colorectal cancers.^270^ Slow-growing tumors are common in this age group. Response to cancer treatment is better related to the physiological status rather than the age.
- **Osteoarthritis** is a very common chronic disorder among older adults and a frequent cause of chronic pain and disability.^271^ The occurrence of osteoarthritis is higher among women than men. Obesity is a risk factor for osteoarthritis, with increasing rate of severe hip and knee arthritis. Osteoarthritis treatments include expensive joint replacement surgery, in addition to intensive rehabilitative treatments. Lower back pain is a common symptom, and its cause is often multifactorial.^265^
- **Diabetes** rates are on the rise in the aging population. Diabetes is a strong risk factor for cardiovascular disease in older adults.^272^ It is also related to peripheral arterial disease and peripheral neuropathy, causing diabetic foot ulcers and amputations.
- **Osteopenia/Osteoporosis.** Osteopenia is normal loss of bone density upon aging. Older adults frequently suffer from osteoporosis, a harsher deterioration of bone density.^273^ Osteoporosis is associated with an increased rate of bone fractures. Calcium and vitamin D supplementation may be efficient in preventing osteoporosis and bone fractures.
- **Sarcopenia** is an age-related gradual loss of muscle mass and strength, a type of muscle atrophy primarily caused by the natural aging process. It is one of the most important causes of functional decline and loss of independence in older adults. Being physically inactive and eating an unhealthy diet can contribute to the disease.^274^
- **Chronic obstructive pulmonary disease (COPD)** is a common age-related disease. It is typified by a reduction of airflow into the lungs due to the inflammation of airways, thickening of the lungs lining, and an overproduction of mucus in the air tubes.^275^
- **Cognitive decline** produces mild short-term memory loss, difficulty finding words, and slower processing, which are all normal features of aging. Deviations from normal brain aging may lead to dementia, manifesting as memory loss, mood changes, confusion, communication difficulties, or deprived judgment.^276^ Rates of dementia rise with age. Alzheimer’s disease is the most common cause of dementia,^277^ but a number of other disorders such as vascular dementia, Lewy body dementia, frontotemporal disorders, Huntington’s disease, and Parkinson’s disease can trigger it as well.

### 2.8 Brain Aging

The brain is remarkably sensitive to the effects of aging, displaying as changes in structure and cognitive capacity, as well as increased risk for developing certain neurological disorders.^278,279^ Brain health refers to the maintenance of brain functions in several aspects: (i) cognitive health—the ability to adequately think, learn, and remember; (ii) motor function—the ability to control movements and balance; (iii) emotional health—the ability to interpret and respond to emotions; (iv) tactile function—the ability to feel and respond to sensations of touch, including pressure, pain, and temperature.^280^

At the molecular level, brain aging, similarly to all other organ systems, is characterized by changes in gene expression, epigenetic modifications, and alterations in protein synthesis and turnover. It is also associated with the accumulation of toxic protein aggregates, such as β-amyloid and tau, which can disrupt neuronal function and contribute to the development of neurodegenerative diseases.^12,17^ At the cellular level, brain aging is characterized by the accumulation of cell damage, including oxidative stress, DNA damage, and protein misfolding. This damage can lead to the dysfunction and death of brain cells, including neurons and glia. Studies have shown that dendritic arbors and spines decrease in size and/or number in cortex as a result of aging.^281,282^ Aging also sets off a decline in the regenerative capacity of brain cells, such as decreased neurogenesis and oligodendrogenesis.^14,15^

At the system level, brain aging includes changes in brain connectivity and function such as alterations in neural activity, neurotransmitter function, and white matter integrity. Aging is associated with a decline in the function of essential neurotransmitter systems such as dopamine and acetylcholine, which can lead to cognitive impairment. Brain aging is associated also with changes in brain structure, such as the loss of gray matter volume and changes in white matter microstructure.^10,283−285^ At the organismal level, brain aging is associated with declines in cognitive function, sensory function, and motor function. Age-related changes in the cardiovascular system, immune system, and endocrine system can also impact brain function and contribute to age-related neurodegenerative diseases.^17,286^

Hallmarks of aging, including mitophagy, cellular senescence, genomic instability, and protein aggregation, have been related to the age-associated neurodegenerative and cerebrovascular disorders.^18^ Furthermore, the most frequent neurodegenerative diseases share the common attribute of protein aggregation. The aggregation of senile plaques containing amyloid-β peptide and the formation of intraneuronal tau containing neurofibrillary tangles in Alzheimer’s disease and the accumulation of misfolded α-synuclein in Parkinson’s disease are major pathogenic aspects of these diseases.^287^ Protein aggregation is also a feature of amyotrophic lateral sclerosis and frontotemporal lobar dementia.^288^

Brain tissues comprise primarily postmitotic cells, including neurons and oligodendrocytes, which are sensitive to age-related alterations such as DNA damage or methylation. Indeed, Parkinson’s disease patients have been reported to consistently exhibit DNA methylation patterns associated with advanced aging.^289^ Advanced aging has been also related to enhanced mitochondrial dysfunction and damage, thus promoting neurodegeneration via the production of ROS and the advancing neuroinflammation.^17^

In addition to the most common age-associated neurodegenerative diseases such as Alzheimer’s and Parkinson’s diseases and stroke, others included are age-related macular degeneration associated with blurred or distorted vision; multiple sclerosis associated with myelin damage, which disturbs the information flow within brain, and between brain and body; amyotrophic lateral sclerosis (Lou Gehrig’s disease) affecting motor neurons thus causing loss of muscle control; Huntington’s disease associated with involuntary movements, difficulty with coordination, and changes in mood and behavior; and various kinds of dementias including Lewy bodies dementia characterized by the presence of abnormal protein deposits in the brain, which causes changes in attention and alertness, visual hallucinations, and movement disorders, and vascular dementia associated with damage to the blood vessels that supply blood to the brain, which causes memory loss, difficulty with decision-making, and changes in mood and behavior.^17,283,290,291^

**Figure 7:**
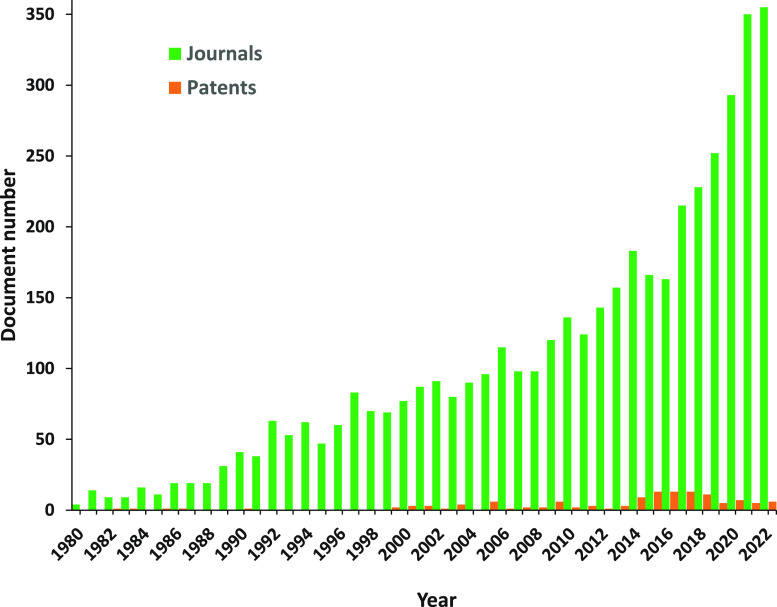
Yearly growth of the number of documents related to brain aging in the CAS Content Collection.

There is a steady, nearly exponential growth of the number of journal publications related to brain aging in the CAS Content Collection over time, remarkably intense in the last two years (Figure 7), a sign of the enhanced scientific interest in this area. At the same time, patenting activity is low, probably awaiting the knowledge accumulation reaching a critical level.

### 2.9 Skin Aging

Skin aging is one of the most studied aspects of aging because it is visible and can affect a person’s appearance, which can have significant social and psychological effects. Aging of the skin can lead to changes in skin texture, color, and elasticity, which can affect how people look and feel about themselves. Furthermore, the skin plays an important role in protecting the body from environmental factors, such as UV radiation and pollution. It also prevents excessive water loss and the entry of toxic substances and pathogens into the environment. Upon aging, the skin’s ability to perform these functions can decrease, which can have negative effects on overall health.

As the largest organ of the body exposed to the external environment, the skin endures both intrinsic and extrinsic aging factors with extrinsic aging prompted by environmental impacts and overlaying the effects of temporal aging. Intrinsic aging is a physiological process that results in several phenotypes such as, but not limited to, wrinkling, pigmentation, telangiectasis, and gradual dermal atrophy,^292−298^ while extrinsic aging is provoked by exterior environment and behavioral factors such as air pollution, tobacco smoking, inadequate nutrition, and sun exposure, causing wrinkles, elasticity loss, as well as rough-textured appearance.^292,293^ Particularly, long-term exposure to solar UV radiation is the prime factor of extrinsic skin aging referred to as photoaging.^293^

Skin aging is accompanied by phenotypic changes in cutaneous cells along with structural and functional alterations in extracellular matrix components such collagen, elastin and proteoglycans, which are required to afford tensile strength, elasticity, and moisture to the skin.^299,300^ This can result in the appearance of fine lines and wrinkles, sagging skin, and a loss of facial volume. In addition, skin aging is characterized by a decrease in the level of production of hyaluronic acid, a substance that helps to maintain skin hydration and suppleness. Other intrinsic factors that contribute to skin aging include genetic inheritance, slower cell turnover, and hormonal changes, including estrogen, progesterone, and testosterone decrease, which can affect the skin structure. which can lead to a loss of skin elasticity and changes in skin cell metabolism. Additionally, changes in skin microbiota, the collection of microorganisms that live on our skin, can contribute to skin aging and the development of aging-associated skin diseases.^299^

Extrinsic factors that can contribute to skin aging include exposure to ultraviolet (UV) radiation, cigarette smoke, pollution, and a poor diet. UV radiation from the sun is a major contributor to skin aging, causing damage to the skin cells and breaking down collagen and elastin fibers. This can result in the development of age spots, a rough texture, and uneven skin tone. Additionally, exposure to cigarette smoke and pollution can cause oxidative stress, leading to inflammation and damage to skin cells. A diet that is high in sugar, processed foods, and unhealthy fats can lead to inflammation, which can also accelerate the aging process.^301,302^

Macrophages are the most abundant immune cell type in the skin and are vital for skin homeostasis and host defense.^303^ However, they have also been associated with chronic inflammation upon aging. It has been suggested that age-modified skin macrophages may promote adaptive immunity exacerbation and exhaustion, facilitating the development of proinflammatory pathologies, including skin cancer.^303^

While the intrinsic and extrinsic aging factors are both related to phenotypic changes in dermal cells, the most significant structural changes take place in the extracellular matrix (ECM) of dermis, in which collagens, elastin, and proteoglycans impart tensile strength and hydration. The utmost longevity of these biomolecules, relative to the intracellular proteins, exposes them to accumulated damage, which in turn affects their capability to provide mechanical properties and to manage tissue homeostasis.^304−308^ Thus, at variance with the intracellular proteins, the half-lives of which are measured in hours or at most days,^306^ many ECM proteins exhibit half-lives measured in years. For instance, human skin and cartilage collagens types I and II have half-lives of about 15 and 95 years,^309^ while the half-lives of elastin fibers is equal to^304^ or many times longer than average human life.^310,311^ Therefore, in humans, ECM proteins are required to function for long years, during which time they are at risk of accumulating damage via glycation,^312^ calcium and lipid accumulation,^313,314^ and alterations of aspartic acid residues.^305,315^ In turn these events have a profound effect on the mechanical properties of ECM proteins.^316^

Various molecular models are proposed to rationalize the molecular basis of skin aging, mostly including the overall recognized aging mechanisms such as cellular senescence, telomere shortening, decrease in cellular DNA repair capacity and point mutations of extranuclear mitochondrial DNA, oxidative stress, chromosomal abnormalities, gene mutations, and chronic inflammation (inflammaging).^316^

While skin aging is a natural process that cannot be completely prevented, there are steps that can be taken to slow the process and maintain healthy skin. These include protecting the skin from UV radiation by wearing protective clothing and using sunscreen, avoiding smoking and exposure to pollution, and maintaining a healthy diet and lifestyle. Additionally, skincare products that contain ingredients such as retinoids, antioxidants, and hyaluronic acid can help decrease the appearance of fine lines and wrinkles, improve skin texture and tone, and enhance hydration. Generally, the strategies for treating skin aging include the common antiaging approaches: stem cell therapy, hormone replacement therapy, telomere modification, diet restriction, and also antioxidant, retinoid, and anti-inflammaging treatments.^316^

In addition to its social and health-related implications, skin aging is also an area of interest for the cosmetics and skincare industries. There is a large market for antiaging skincare products, and research into the underlying mechanisms of skin aging can help to develop new and more effective products.

## 3 Landscape View of Aging Hallmarks and Factors: Insights from the CAS Content Collection

The CAS Content Collection^28^ is the largest human-compiled collection of published scientific information, which represents a valuable resource to access and keep up to date on the scientific literature all over the world across disciplines including chemistry, biomedical sciences, engineering, materials science, agricultural science, and many more, thus allowing quantitative analysis of global research publications against various parameters including time, geography, scientific area, medical application, disease, and chemical composition. Currently, there are over 500 000 scientific publications (mainly journal articles and patents) in the CAS Content Collection related to aging physiology and antiaging strategies. There is a steady growth of these documents over time, especially intense in the past decade (Figure 2).

**Figure 8:**
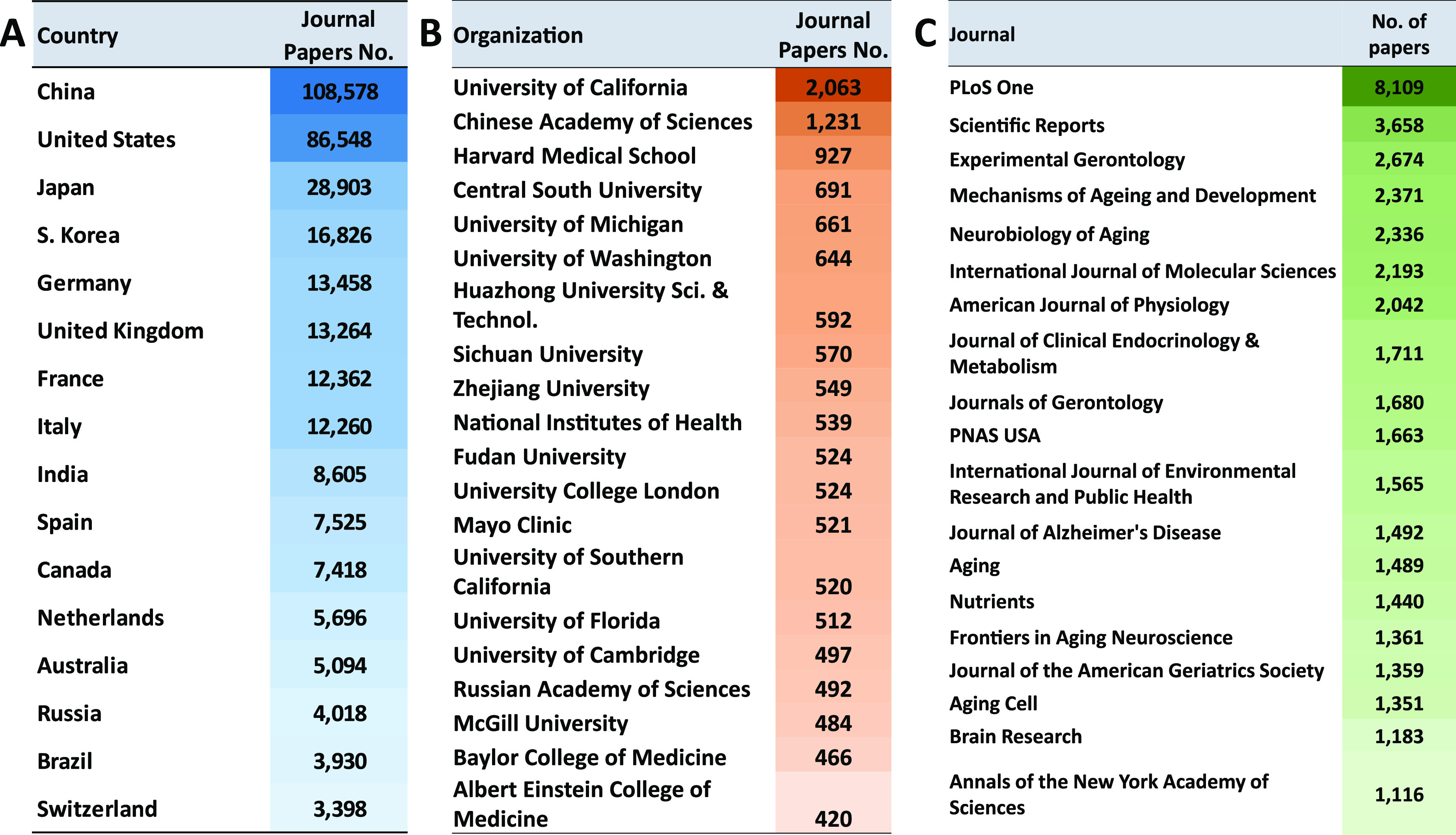
Top countries (A), organizations (B), and scientific journals (C) publishing articles related to aging mechanisms and antiaging strategies.

China, the United States, Japan, and South Korea are the leaders with respect to the number of published journal articles (Figure 8A) related to aging physiology and mechanisms. University of California and the Chinese Academy of Sciences are the leaders with the largest number of published articles in scientific journals (Figure 8B). The journal *PLoS One* is the distinct leader publishing the highest number of articles related to the physiological mechanisms of aging (Figure 8C).

**Figure 9:**
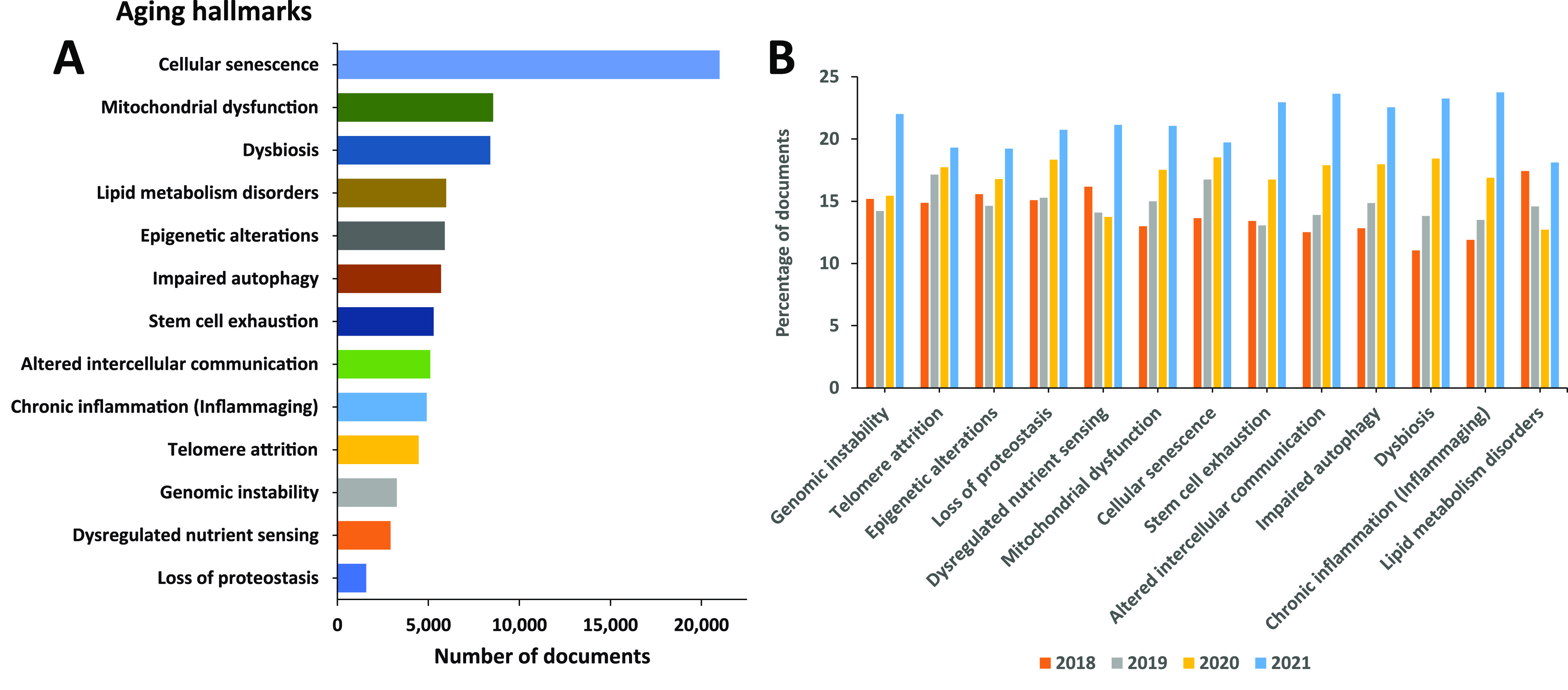
Hallmarks of aging explored in the scientific publications: (A) number of publications exploring hallmarks of aging; (B) trends in number of publications exploring hallmarks of aging during the years 2018–2021.

We further explored the distribution and trends in published documents dealing with the various hallmarks of aging (Figure 9). The cellular senescence is clearly the aging attribute attracting the most attention (Figure 9A). This should come as no surprise since cellular senescence is the aging denominator closely related to all other aging features (Figure 4). It has been also connected to multiple age-related disorders, including cancer, diabetes, osteoporosis and osteoarthritis, cardiovascular disease, stroke, Alzheimer’s disease and other dementias; furthermore, it has also been linked to deteriorations in eyesight, mobility, and cognitive capability.^169^ With respect to the annual trends in the aging hallmark related publications, steady annual growth has been seen in those related to stem cell exhaustion, altered intercellular communication, impaired autophagy, and especially dysbiosis (Figure 9B). Indeed, the extreme significance of gut microbiome in multiple aspects of human health is recently becoming well renowned and a hot topic in scientific research.^317^ Substantial data suggest that the gut microbiome plays a role in virtually all physiological processes in the human body, including metabolism and immune homeostasis. Alterations to these processes can disturb the balance in the microbiome (a process termed dysbiosis) and trigger a range of pathological processes fundamental to mental health, metabolism, including multiple age-related diseases.^318^

**Figure 10:**
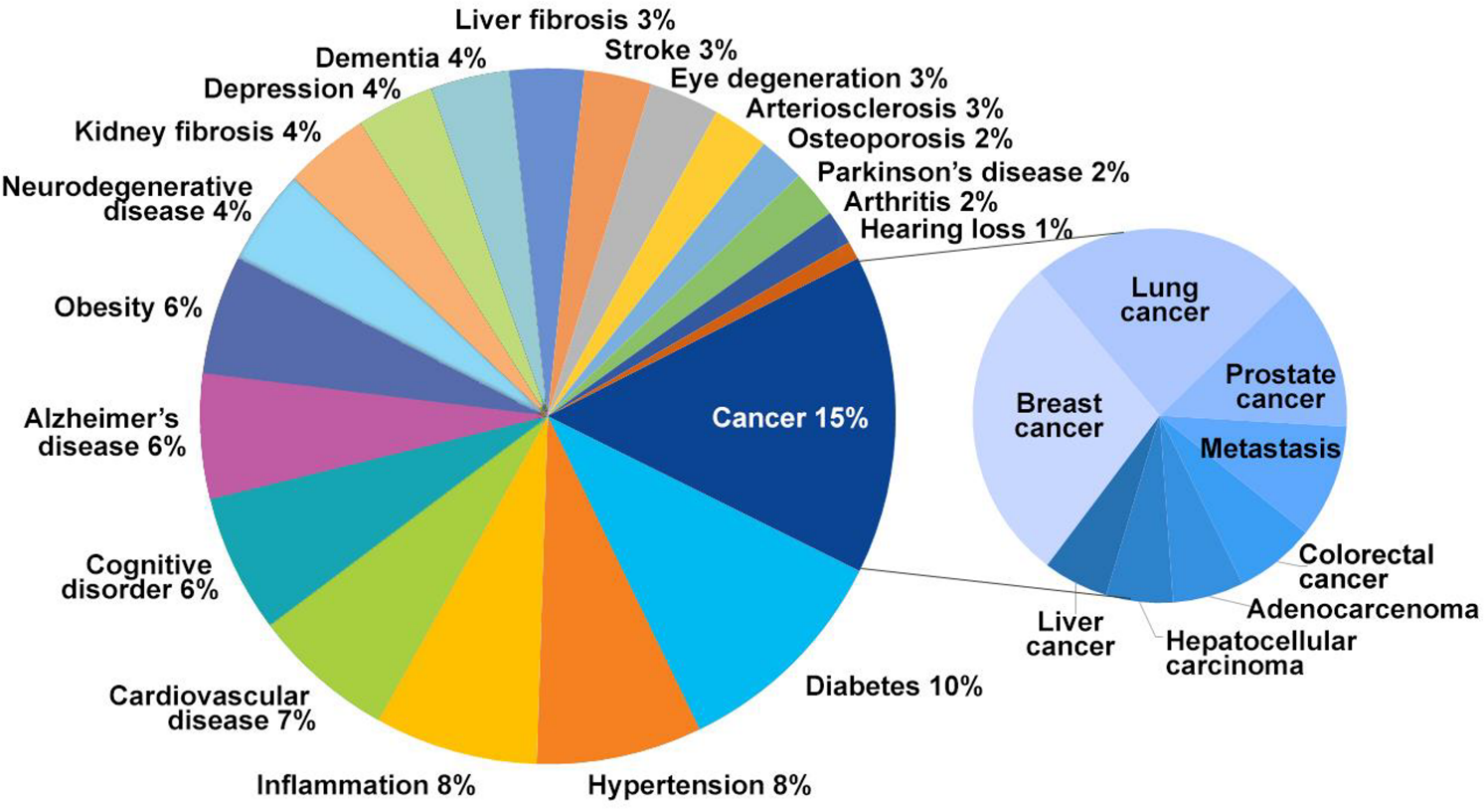
Distribution of documents in the CAS Content Collection related to age-associated diseases.

A wide collection of diseases are associated with aging. Figure 10 illustrates the distribution of documents in the CAS Content Collection related to such age-associated pathologies. Among these major diseases are cancer, diabetes, and hypertension. Inflammation, cardiovascular disease, and cognitive disorders are also highly represented (Figure 10).

**Figure 11:**
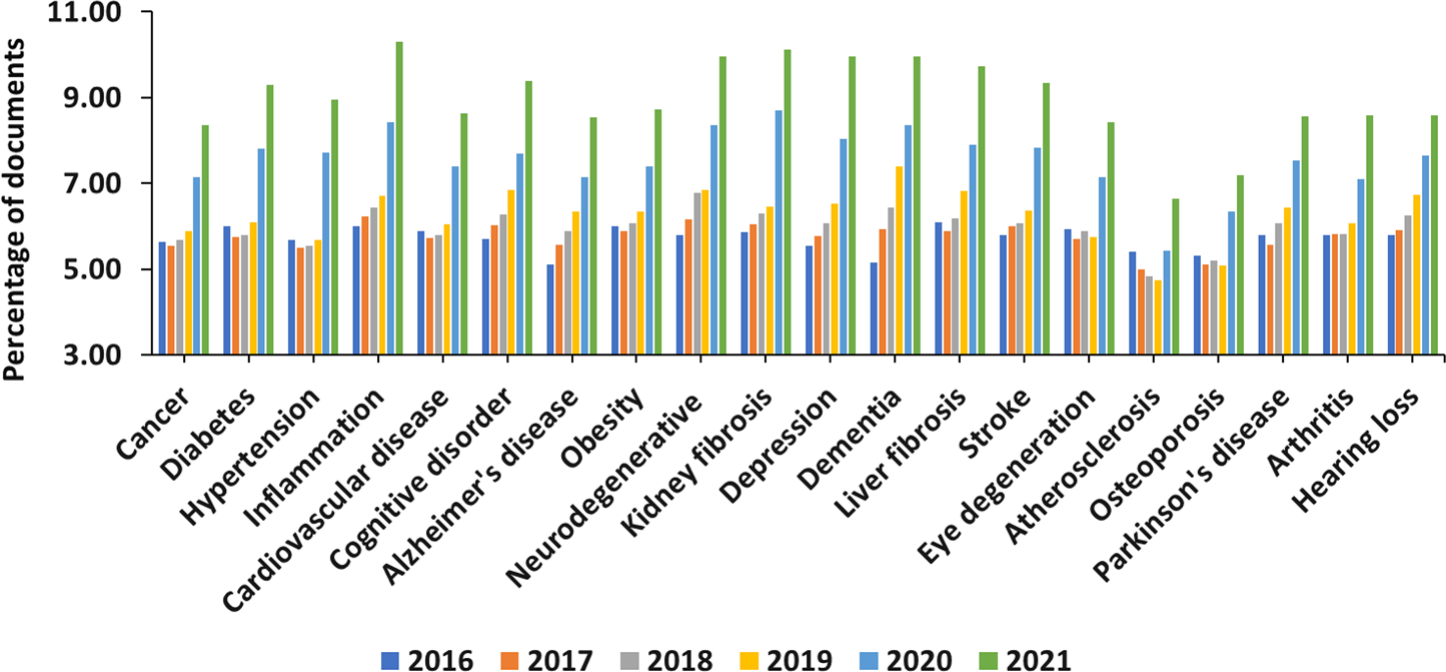
Annual growth of number of documents related to age-associated diseases.

Figure 11 presents the annual trend in the number of documents related to age-associated diseases. A steady growth in the number of recent publications has been documented with respect to inflammation and neurodegenerative diseases including dementia and depression. Indeed, chronic low-grade inflammation (inflammaging) has been identified as playing an increasingly important role in the rate of aging and has recently emerged as a challenging and promising new domain of aging-related research.^319^ Neurodegenerative diseases, although originating from different primary causes, all share a hallmark of neuroinflammation.^320^

**Figure 12:**
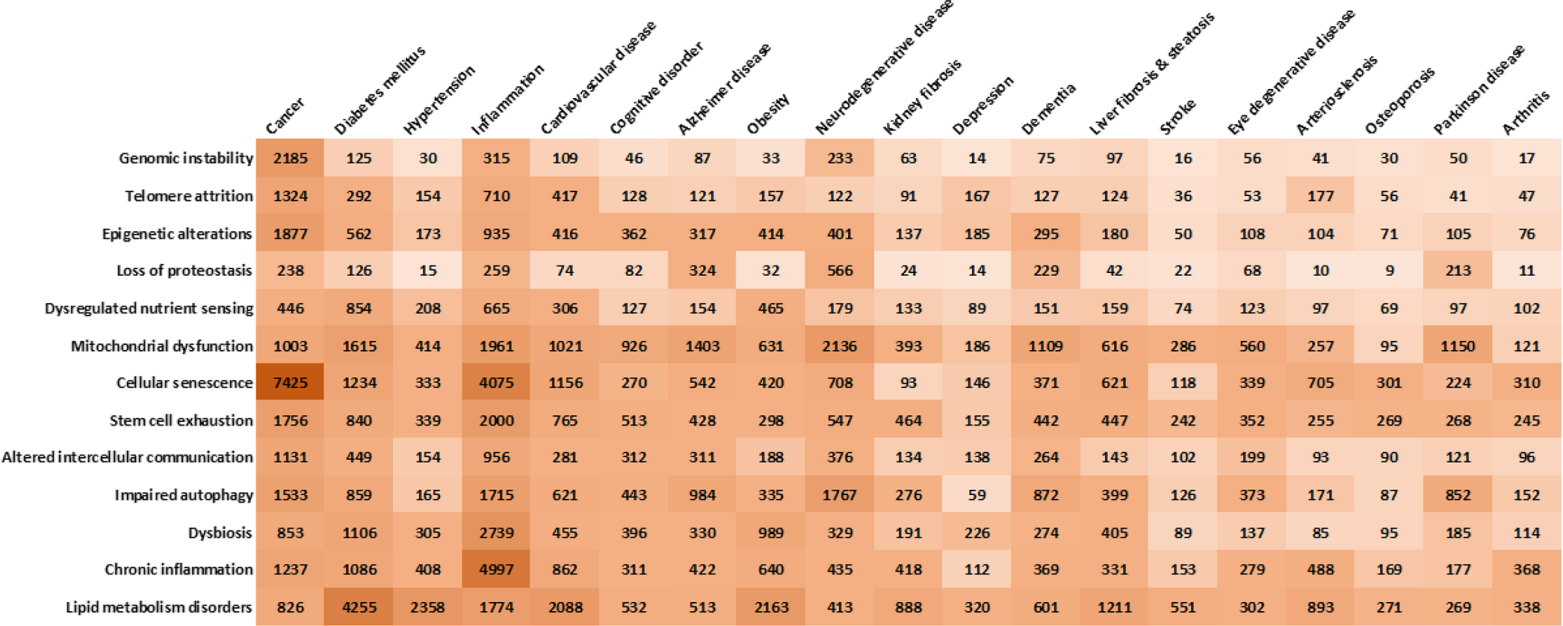
Correlation of the number of documents related to the hallmarks of aging with age-related diseases.

**Figure 13:**
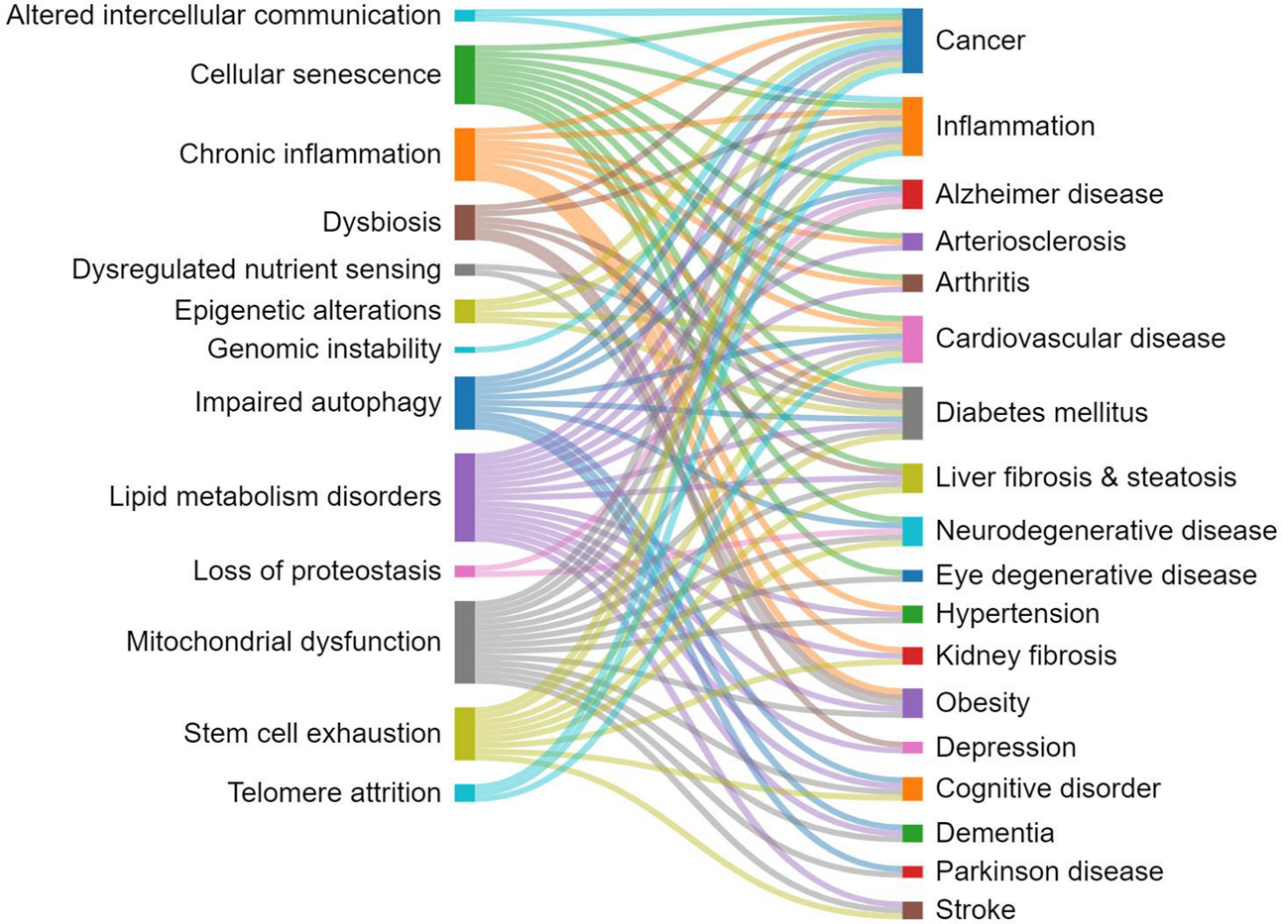
Relationship between the hallmarks of aging and the age-related diseases as revealed by the number of corelated documents (Figure 12).

We explored the correlations between the aging hallmarks and the age-related diseases, as reflected in the number of documents in the CAS Content Collection (Figures 12 and 13). Generally, cellular senescence, mitochondrial dysfunction, lipid metabolism disorders, and inflammaging appear as related to multiple pathologies.

Some particular correlations are noteworthy:

- There is a strong correlation between documents related to cellular senescence and cancer, according to the CAS Content Collection. Cellular senescence is a state of a cell cycle arrest, so the entry of cells into senescence can act as a barrier to tumorigenesis thus being of special interest for anticancer therapies. It has been demonstrated however that, in certain conditions, malignant and nonmalignant senescent cells can develop protumorigenic properties and eventually trigger tumor relapse, evidencing contrasting roles of senescent cells in cancer still remaining to be explored.^321−323^
- The strongest correlation between diabetes mellitus and aging hallmarks is with the lipid metabolism disorders, according to the CAS Content Collection documents number. Glucose and lipid metabolism are correlated in multiple ways.^324^ One of the notable manifestations of this correlation is diabetic dyslipidemia, with both being well established cardiovascular risk factors. The link between glucose and lipid metabolism is in fact rather complex with both lipids and glucose playing important roles in energy metabolism.^324−326^
- Hypertension–lipid metabolism disorders correlation: It has been reported that both hypertension and aging are associated with higher lipid peroxidation.^327^ Aging is additionally associated with an increase in lipid peroxidation in cardiac muscle.^328^
- Inflammation–cellular senescence correlation: Aging is characterized by systemic chronic inflammation, linked to cellular senescence, immunosenescence, and age-related organ dysfunction. Senescence-associated secretory phenotype (SASP) factors secreted by senescent cells promote chronic inflammation. Meanwhile, chronic inflammation accelerates the senescence of immune cells, resulting in an inability to clear inflammatory factors, which creates a malicious cycle of inflammation and senescence.
- Altogether, there is significant correlation between cellular senescence and the majority of age-related diseases.^329^ The disadvantages of senescence seem to be in, first, causing a loss of tissue-repair capacity because of cell cycle arrest in progenitor cells and, second, in producing proinflammatory molecules in the senescence-associated secretory phenotype (SASP). Substantial pool of information about senescence in cells has been acquired recently; however, it is still poorly understood.
- Cognitive impairment–mitochondrial dysfunction correlation: The brain profoundly depends on mitochondria to produce energy, in order to maintain essential bodily functions. Upon aging, damaged mitochondria accumulate. They produce insufficient ATP and excessive ROS. It has been recently reported that mitochondria at dysfunctional synapses do not meet the energetic need and potentially trigger age-related cognitive impairment.^330,331^
- Alzheimer disease–mitochondrial dysfunction correlation: Alzheimer’s disease is the most frequent source of age-related neurodegeneration and cognitive impairment. A growing body of evidence implicates mitochondrial dysfunction as a common pathogenic mechanism involved in many of the features of the Alzheimer’s patients brain, such as formation of amyloid plaques and neurofibrillary tangles.^332^
- Altogether, there is significant correlation between mitochondrial dysfunction and the majority of age-related diseases including diabetes, inflammation, obesity, neurodegenerative disorders, cardiovascular diseases, and cancer.^333^ Mitochondria are vital in regulation of energy and metabolic homeostasis. Proper mitochondrial functions, including cellular energy production and control of oxidative stress, are in strong relation with the accurate performance of brain, cognition, and the overall health.^334^
- Liver fibrosis–lipid metabolic disorders correlation: Liver plays a key role in lipid metabolism; therefore alterations in hepatic lipid metabolism can be a factor in development of chronic liver disease. Furthermore, chronic liver disease can impact hepatic lipid metabolism causing alterations in circulating lipid levels contributing to dyslipidemia.^335^ Likewise, the liver plays an essential role in lipid metabolism, certain steps of lipid synthesis, and transport. Therefore, abnormal lipid profiles and liver dysfunctions are expectedly closely correlated.^336^
- Altogether, there is significant correlation between lipid metabolic disorders and the majority of age-related diseases.^337^ Upon aging, body fat builds up with changes in the lipid metabolism. Considering lipid metabolism, excess body fat with enhanced lipotoxicity triggers various age-related diseases, including cardiovascular disease, cancer, arthritis, diabetes, and Alzheimer’s disease. Progress in lipidomic techniques has identified alterations in lipid profiles associated with aging. Lipid accumulation and impaired fatty acid processing are associated with pathophysiological aging phenotypes. Although it is still not well-known how lipid metabolism is regulated upon aging, data suggest a dynamic role for lipid metabolism in signaling and gene expression regulation.^337,338^

## 4 Clinical Trials

A selection of clinical trials focusing on the exploration of the hallmarks and mechanisms of aging are showcased to reveal not only the diversity of trials currently in the pipeline but also areas of completed research (Table 4). Clinical trials focusing on the aging brain and health, such as trials researching neurodegeneration, cognitive decline, attention, thinking and planning, bladder control, and the gut–brain axis, are well represented in the clinical pipeline and highlighted in Table 4. Also highlighted are clinical trials examining inflammation, stress hormones, skeletal muscle, immune health, and heart health with respect to aging.

**Table 4:** Exemplary Clinical Trials Exploring the Hallmarks of Aging and Related Mechanisms

title	aging mechanism explored	status	sponsor, location	NCT number
Exploring the Gut-Brain Axis in Aging and Neurodegeneration	Aging effects on the gut microbiome	Recruiting	IRCCS San Camillo, Italy	NCT05934188
MicroRNA Regulation of Chronic Inflammation During Aging	The health of aging immune cells in the blood and how these cells affect inflammation and health	Recruiting	University of Utah, USA	NCT05392582
Tau Pet Imaging in the Aging Brain Cohort Dedicated to Diversity Study	Mechanisms and distinctions of age-related cognitive decline and that of preclinical Alzheimer’s disease	Recruiting	University of Pennsylvania, USA	NCT05393388
Cholinergic Mechanisms of Attention in Aging	Cholinergic mechanisms of attention in aging	Recruiting	Vanderbilt University Medical Center, USA	NCT04756232
Investigation of Brain Mechanisms Involved in the Urinary Continence Mechanism Associated With Aging	Define the brain’s key structures, functional activity, and mechanisms involved in normal bladder control and the aging bladder	Recruiting	University of Pittsburgh, USA|National Institute on Aging, USA	NCT04599088
Neurobiological Mechanisms of Aging and Stress on Prospective Navigation	Neural mechanisms of prospective/future goal-directed navigational planning in aging	Recruiting	Georgia Institute of Technology, USA|National Institute on Aging, USA	NCT03896529
Physical Resilience: Indicators and Mechanisms in the Elderly (PRIME) Collaborative Phase 2	Identify important predictors and characteristics of physical resilience	Active, not recruiting	Duke University, USA|National Institute on Aging, USA	NCT04235309
Patterns of Natural Aging and the Role of Senescence Registry	Measured biomarkers of aging/senescence to build computational models of aging to better understand the role of senescence in aging-related functional decline	Completed	University of North Carolina, USA	NCT05123859
Biological Aging of Skeletal Muscles in Humans	Mechanisms involved in the biological aging of skeletal muscle in the elderly	Completed	University Hospital of Saint Etienne, France	NCT02675192
Association Between Telomere Length and Risk of Acute Coronary Syndrome	Studied whether mtDNA copy numbers in peripheral blood leukocytes could be used as a risk predictor for acute coronary syndrome	Completed	Air Force Military Medical University, China	NCT02775279
Age-Related Changes in Stress Hormone Regulation	Physiological processes involved in aging-related deficits in stress hormone regulation	Completed	U.S. Department of Veterans Affairs, USA	NCT00018369

## 5 Outlook and Perspectives

Aging is generally defined as the accumulation of detrimental changes taking place in cells and tissues with advancing age, which brings about the increased risk of disease and death. The emerging standpoint defines aging as a particularly complex multifactorial process. Research on aging mechanisms focuses on understanding the underlying biological processes that contribute to the aging of living organisms. This field of study aims to uncover the cellular, molecular, and genetic factors that drive aging as well as the interactions between these factors. Key outlines and perspectives in the research of aging mechanisms include the following:

- Cellular senescence: investigating the role of senescent cells in aging and age-related diseases; understanding the triggers that lead to cells entering a senescent state and their impact on tissue function; exploring therapies to remove or reduce senescent cells (senolytics) to mitigate age-related decline.
- Telomere biology: understanding the role of telomeres in cellular aging and how their shortening contributes to aging; researching telomerase and its potential as a target for antiaging interventions.
- Epigenetics: studying changes in gene expression patterns with age and their impact on cellular function; exploring the role of DNA methylation, histone modifications, and noncoding RNAs in age-related changes.
- Mitochondrial function: investigating the role of mitochondrial dysfunction in aging and age-related diseases; studying the impact of mitochondrial DNA mutations and oxidative stress on cellular aging.
- Proteostasis: understanding how protein homeostasis is maintained and how protein misfolding and aggregation contribute to aging; researching proteostasis mechanisms, including chaperones and the ubiquitin–proteasome system.
- Cellular energetics: exploring the role of cellular energy production and metabolism in aging; studying the impact of nutrient sensing pathways, such as mTOR and AMPK, on aging.
- Immune system aging: investigating age-related changes in the immune system, such as immunosenescence and inflammaging; understanding how immune responses decline with age and contribute to susceptibility to infections and chronic diseases.
- Genetic influences: identifying genetic factors that influence longevity and age-related diseases through genome-wide association studies and other approaches; studying genetic variants and genes associated with exceptional longevity.
- Interventions and therapies: evaluating potential antiaging interventions, such as caloric restriction, pharmacological agents, and senolytics; exploring the potential of gene editing technologies (e.g., CRISPR) for age-related diseases.
- Systems biology and network analysis: applying systems-level approaches to understand the interconnectedness of aging mechanisms; using network analysis to identify key regulators and pathways involved in aging.
- Comparative biology: studying aging mechanisms across different species to identify conserved pathways and potential targets for interventions.
- Artificial intelligence and data analysis: utilizing AI and big data analysis to uncover patterns and relationships in complex aging data sets.

Research in the field of aging mechanisms is interdisciplinary, involving genetics, cell biology, biochemistry, immunology, neuroscience, and other disciplines. The goal is to gain a comprehensive understanding of the processes that drive aging and develop targeted interventions to promote healthy aging and extend lifespan.

The research of human aging faces several roadblocks and challenges that can hinder progress and understanding. Some of the key roadblocks include the following:

- Complexity of aging processes. Aging is a multifaceted and complex phenomenon influenced by numerous interacting factors, including genetics, epigenetics, cellular biology, and environmental influences. Understanding the precise mechanisms and interactions involved is challenging.
- Longitudinal studies. Studying aging requires long-term, large-scale longitudinal studies to track individuals over extended periods of time. Conducting such studies can be resource-intensive and time-consuming.
- Ethical considerations. Some research involving aging may raise ethical concerns, especially when it comes to potential interventions or the use of certain experimental methods on human subjects.
- Lack of standardization. Aging research often involves a wide variety of methodologies, biomarkers, and outcome measures. The lack of standardization makes it challenging to compare and integrate findings from different studies.
- Limited human-specific models. While research in model organisms provides valuable insights, humans have unique characteristics that cannot be entirely replicated in animal models.
- Limited funding. Aging research may not receive as much funding and attention as other areas of research, despite its significant societal impact.
- Time scale. Studying aging requires long observation periods, which can be a limiting factor in rapidly advancing scientific research.
- Data interpretation. Analyzing complex data from aging studies can be challenging, and there is a need for sophisticated statistical and computational approaches.
- Gender and diversity bias. Historically, many aging studies have been biased toward male participants and lacked diverse representation, which may impact the generalizability of results.
- Interdisciplinary collaboration. Aging research requires collaboration across various disciplines, and fostering effective communication between experts from different fields can be challenging.
- Public perception. Some aspects of aging research, such as life extension technologies, face skepticism or resistance from the public, leading to limited support and funding.
- Translation to clinical applications. Translating basic research findings into clinical applications and interventions that effectively promote healthy aging remains a significant challenge.

Despite these roadblocks, ongoing efforts, technological advancements, and interdisciplinary collaborations are steadily advancing our understanding of human aging. Addressing these challenges will be critical in developing strategies to enhance healthy aging, prevent age-related diseases, and improve the overall quality of life for the aging population.
